# Bone, Brain, Heart study protocol: A resilient nested, tripartite prospective cohort study of the role of estrogen depletion on HIV pathology

**DOI:** 10.1371/journal.pone.0272608

**Published:** 2022-08-03

**Authors:** C. Christina Mehta, Kimberly S. Hagen, Anna A. Rubtsova, Cecile D. Lahiri, Vasiliki Michopoulos, Caitlin A. Moran, Lisa B. Haddad, Kehmia Titanji, Lauren F. Collins, Arshed A. Quyyumi, Gretchen Neigh, Leslee J. Shaw, M. Neale Weitzmann, Lance Waller, Ighovwerha Ofotokun

**Affiliations:** 1 Division of Infectious Diseases, Department of Medicine, Emory University School of Medicine, Atlanta, GA, United States of America; 2 Department of Behavioral, Social, and Health Education Sciences, Rollins School of Public Health, Emory University, Atlanta, GA, United States of America; 3 Grady Infectious Diseases Program, Grady Health System, Atlanta, GA, United States of America; 4 Department of Psychiatry and Behavioral Sciences, Emory University School of Medicine, Atlanta, GA, United States of America; 5 Center for Biomedical Research, Population Council, New York, NY, United States of America; 6 Division of Endocrinology and Metabolism, Department of Medicine, Emory University School of Medicine, Atlanta, GA, United States of America; 7 Division of Cardiology, Department of Medicine, Emory University School of Medicine, Atlanta, GA, United States of America; 8 Department of Anatomy and Neurobiology, Virginia Commonwealth University School of Medicine, Richmond, VA, United States of America; 9 Blavatnik Women’s Health Research Institute, Icahn School of Medicine at Mount Sinai, New York, NY, United States of America; 10 Atlanta Veterans Affairs Medical Center, Decatur, GA, United States of America; 11 Department of Biostatistics and Bioinformatics, Rollins School of Public Health, Emory University, Atlanta, GA, United States of America; GERMANY

## Abstract

**Purpose:**

We describe the rationale for and design of an innovative, nested, tripartite prospective observational cohort study examining whether relative estrogen insufficiency-induced inflammation amplifies HIV-induced inflammation to cause end organ damage and worsen age-related co-morbidities affecting the neuro-hypothalamic-pituitary-adrenal axis (Brain), skeletal (Bone), and cardiovascular (Heart/vessels) organ systems (BBH Study).

**Methods:**

The BBH parent study is the Multicenter AIDS Cohort/Women’s Interagency HIV Study Combined Cohort Study (MWCCS) with participants drawn from the Atlanta MWCCS site. BBH will enroll a single cohort of n = 120 women living with HIV and n = 60 HIV-negative women, equally distributed by menopausal status. The innovative multipart nested study design of BBH, which draws on data collected by the parent study, efficiently leverages resources for maximum research impact and requires extensive oversight and management in addition to careful implementation. The presence of strong infrastructure minimized BBH study disruptions due to changes in the parent study and the COVID-19 pandemic.

**Conclusion:**

BBH is poised to provide insight into sex and HIV associations with the neuro-hypothalamic-pituitary-adrenal axis, skeletal, and cardiovascular systems despite several major, unexpected challenges.

## Introduction

Funded in 2018 (U54AG062334), the Specialized Centers of Research Excellence on Sex Differences (SCORE) at Emory University focuses on the HIV-host pathogen interaction as a model for probing the influence of sex as a biologic variable (SABV) on the pathology and pathogenesis of infectious diseases. Globally, over 50% of the 37.7 million people living with HIV are women [1] and HIV infection remains the leading cause of disease and death for women of childbearing age worldwide [2–4]. Even when controlled with antiretroviral therapy (ART), HIV increases the risk for developing age-related comorbidities in women [5–7]. Emerging data suggest that the modifying effects of sex on HIV associated age-related co-morbidities is particularly pronounced in certain end-organs, including the central nervous [8–11], skeletal [12, 13], and cardiovascular [14–18] systems.

The Emory SCORE is investigating the effect of relative estrogen insufficiency on the HIV-host interaction in women via the Brain, Bone, Heart (BBH) study, a nested, tripartite sub-study of the Multicenter AIDS Cohort Study (MACS)/Women’s Interagency HIV (WIHS) Study Combined Cohort (MWCCS). BBH is examining the degree to which, if any, estrogen insufficiency-induced inflammation converges with HIV-induced inflammation to cause end organ damage and worsen age-related co-morbidities affecting the neuro-hypothalamic-pituitary-adrenal (neuro-HPA) axis (Brain), skeletal (Bone), and cardiovascular (Heart/vascular) organ systems. Understanding mechanisms underlying ongoing inflammation in virologically suppressed women living with HIV could ultimately lead to novel preventative and therapeutic interventions to limit inflammation and subsequent end organ damage in the women. This manuscript describes the rationale behind BBH, outlines the BBH protocol, and describes protocol adjustments to meet two serious study disruptions: a change in the scope and activities of the parent study and the COVID-19 pandemic.

### Estrogen deficiency and immune activation

Important sex differences exist in the immune response to antigens, including infectious diseases, vaccines, and self-antigens, and estrogen has been shown to exert a potent effect on both the innate and the adaptive immune systems [19, 20]. Clinical evidence of the immunomodulatory effects of endogenous estrogen abounds and includes reduced symptoms of chronic inflammatory diseases including rheumatoid arthritis (RA), inflammatory bowel disease, and multiple sclerosis during pregnancy. By contrast, higher rates of inflammatory bone disease, RA and cardiovascular disease are documented in postmenopausal women. Emerging data indicate that immune effects of estrogen are bimodal, depend on circulating levels, cell type, and are mediated primarily via estrogen receptors (ER)α and ERβ, which are widely distributed in cells and tissues in the body [19]. At high concentrations such as during pregnancy, estrogen inhibits important pro-inflammatory pathways such as tumor necrosis factor (TNF)-α, interleukin (IL)-1β, IL-6, monocyte chemoattractant protein (MCP)-1, inducible nitric oxide synthase (iNOS) expression, production of matrix metalloproteinase (MMPs), and activity of natural killer (NK) cells [21, 22]. Estrogen further stimulates anti-inflammatory pathways such as IL-4, IL-10, transforming growth factor (TGF)-β, tissue inhibitor of MMPs (TIMP), and osteoprotegerin (OPG) [19]. At lower concentrations such as observed in postmenopausal women, estrogen promotes inflammation by stimulating TNF-α, interferon (IFN)-γ, IL-1β, and activity of NK cells [19].

### Immune effects of HIV infection

HIV establishes a chronic and latent infection that is not eliminated by host immune defenses nor completely eradicated by ART. Extensive immune system damage occurs affecting both cellular and humoral immune responses and leads to severe T cell (both CD4+ and CD8+) depletion and B cell exhaustion, predisposing patients to secondary and opportunistic infections. Commonly, untreated HIV infection involves persistent viral replication leading to a gradual loss of CD4+ T cells, and increased immune activation affecting all major immune system cell populations, driving chronic inflammation [23]. HIV denudes the gastrointestinal system, leading to increases in gut permeability and microbial translocation, contributing to systemic inflammation and immune activation [24, 25]. While these effects are ameliorated by successful treatment with ART, they are not completely normalized even in those with virologic suppression [26].

### Brain, bone, and heart/vascular

Emerging data suggest that the modifying effects of sex on HIV associated age-related co-morbidities is particularly pronounced in certain end organs including the central nervous, skeletal, and cardiovascular systems. Exposure to stress and trauma that leads to posttraumatic stress disorders and other stress-related psychiatric conditions are highly prevalent among WLH [8–11, 27], and can result in greater systemic inflammation due to dysregulation of the neuro-HPA axis and over-activation of the sympathetic nervous system [28, 29]. HIV is a risk factor for bone mineral density (BMD) loss with up to 70% of infected individuals osteopenic [30–37] and over 15% osteoporotic [38]; the fracture rate with chronic HIV infection is up to 5-fold higher compared with the general population [17, 18, 39, 40]. Interestingly, BMD loss in the setting of HIV occurs twice as fast among women [12] and HIV status heightens the rise in fracture incidence observed in women following transition to menopause [13]. Lastly, in several cohort studies, WLH had an approximately 3-fold increased risk of myocardial infarction (MI) [14, 17, 18] and an approximately 2-fold increased risk for ischemic stroke, compared with a 1.5-fold increased risk for MI and no increased risk for stroke among men living with HIV [15, 16]. Remarkably, this increased risk among WLH is greatest among young people—women under age 45 living with HIV have an ~5-fold increased risk of MI [14] and an ~4-fold increased risk of ischemic stroke [16] compared with similarly-aged women in the general population.

The identification of causal pathways underlying the influence of sex on the pathology and pathogenesis of chronic HIV and other infections will enable the development of interventions and therapies that take these factors into account. New insights into sex-based effects will play a critical role in the development of more individualized treatment concepts in infectious disease that take into account host factors as well as pathogen diversity and susceptibility. Given the inherent over representation of men in clinical trials and the limited analysis of existing clinical trial data according to sex, focused research on women as an important and underrepresented group in infectious diseases research is even more critical given the global challenges of historic and emerging infectious diseases.

## Methods

### BBH research hypotheses

To examine the causal pathways underlying the influence of sex on the pathology and pathogenesis of chronic HIV infection, BBH is investigating, within the same cohort of women three interlinked Projects: 1) Brain, which hypothesizes that estrogen deficiency promotes inflammation in WLH that is exacerbated by trauma exposure and trauma-related psychophysiological hyperarousal; 2) Bone, which hypothesizes that HIV-induced immunodeficiency and ART-induced inflammation, could exacerbate estrogen deficiency bone loss in older WLH; and 3) Heart/vascular, which hypothesizes that HIV-related inflammation in women worsens endogenous reparative/regenerative processes in the setting of estrogen deficiency and that these have a combined impact on the presence and progression of sub-clinical coronary and carotid artery atherosclerosis. The specific aims of each BBH Project are listed in Table 1. Note that Aim 1 in Project 1 provides data that is used in both Project 2 and Project 3. In addition, Project 2 contains a clinical Aim, as well a related mechanistic experimental Aim, performed in animal models.

**Table 1:** Specific aims for each Brain, Bone, Heart (BBH) study project.

Project	Aim
Project 1 Brain	Aim 1. Define estrogen deficiency at both the systemic and receptor level and evaluate the extent to which global variation in these parameters predicts pro-inflammatory cytokines.
	Aim 2. Determine the extent to which trauma exposure and trauma-related hyperarousal interact with HIV to predict estrogen deficiency and pro-inflammatory cytokines.
	Aim 3 (exploratory). Describe the influence of trauma exposure and estrogen receptor function on inflammation at the molecular level in peripheral blood mononuclear cells in WLH.
Project 2 Bone	Aim 1. Assess the combined impact of HIV/ART and estrogen deficiency on the skeleton of WLH.
	Aim 2*. Assess the collision of estrogen deficiency-induced and ART-induced, inflammatory bone loss in pre-clinical models.
Project 3 Heart/vascular	Aim 1. Study impact of HIV-related changes in regenerative capacity (primary), endothelial function and arterial stiffness (secondary) on prevalent (a) coronary and (b) carotid arterial disease
	Aim 2. Assess the progression of carotid arterial disease by HIV status, using serial MRI over a 2-year period (primary). Secondary Aim: Assess the influence of HIV-related changes in regenerative capacity, inflammation, endothelial dysfunction, arterial stiffness, and estrogen status on progression of carotid artery disease
	Aim 3. Compare the extent of total atherosclerotic plaque volume measured using CCTA by HIV status (primary) and by estrogen status (secondary).
	Exploratory Aim: Investigate high risk coronary plaque characteristics by HIV status

* Conducted in mouse model.

WLH: women living with HIV; ART: antiretroviral therapy; MRI: magnetic resonance imaging; CCTA: coronary computed tomography

### Study design and participants

A unique feature of BBH is that it was designed as a prospective WIHS sub-study and all research participants for the tripartite study were originally intended to be drawn from the Atlanta (Emory University) WIHS site. WIHS was a prospective cohort study of WLH or women at risk for acquiring HIV. In addition to Atlanta, WIHS participants were enrolled at 9 other sites nationally. Every six months WIHS participants underwent a physical and gynecological exam, provided specimens for laboratory analysis, and updated information about their current behaviors, socio-demographics, medications, and physical health status. Additional information about WIHS and MWCC, including cohort descriptions, is described elsewhere [41–44].

Recruitment for BBH started in April 2019 with a (revised) targeted conclusion date of September 2022. During the second year of participant recruitment into BBH, WIHS and MACS, a prospective cohort study of HIV infection among gay and bisexual men, were merged by the NIH to form MWCCS. The transition from a WIHS sub-study to a MWCCS sub-study significantly affected BBH due to revisions to the parent study’s design and implementation, recruitment and retention procedures, revision of the data collection forms that altered both the content and frequency of information and specimens collected from participants, and a reduction from semiannual to annual study visits by participants. Details about the implications of these changes are described below.

#### Sample size

Target enrollment for BBH is n = 120 WLH and n = 60 women at risk for HIV, equally divided by menopausal status (pre-menopause, post-menopause).

Inclusion/exclusion criteria are described in S2 Appendix.

#### Power

Each aim of the three BBH Projects were separately powered (see S1 Table) using the same cohort of n = 180 women.

### Ethical approval and consent

Written informed consent is obtained from all BBH participants. The parent WIHS protocol was initially approved by the Institutional Review Board (IRB) at each WIHS site, including Emory University and, in addition, the BBH protocol was approved by the Emory University IRB and the Grady Health System Research Oversight Committee. After WIHS and MACS merged, the new MWCCS study protocol was approved for all sites by the Johns Hopkins University IRB and prior WIHS and MACS participants were re-recruited and re-consented into MWCCS. Due to challenges with a single IRB for a multi-site study, the MWCCS reverted to individual site IRB approval and informed consent was again obtained from all participants. This slowed BBH recruitment as former WIHS study subjects had to wait for MWCCS recruitment begin and then be re-consented into MWCCS before being consented into BBH.

The protocol for Project 2: Bone Aim 2 (mouse models) was approved by the Emory University Institutional Animal Care and Use Committee (IACUC). See S3 Appendix for more detailed information on steps taken to ameliorate animal suffering and humane endpoints.

### Data collection forms

Prior to study initiation, each project conducted an extensive literature to determine potential confounders. A comprehensive evaluation of proposed elements (exposures, outcomes, confounders/covariates) for data collection from each BBH Project was undertaken to identify inter-Project and WIHS parent study redundancies that would increase participant burden. Questions identified as being common to more than one Project were removed from individual Project forms and asked in a single collective BBH intake form. All BBH forms were assessed for readability of the wording of each question and individual response option, understandability, and consistency, as well as interdependence.

The intense review process was undertaken a second time after WIHS merged into MWCCS in order to ensure that all required BBH data would still be collected by the MWCCS parent study at the same frequency, using the same methods, and the same wording.

### Recruitment and screening

Prior to the MWCCS transition, initial eligibility information from WIHS participants was available for assessment. Use of this information expedited preliminary screening and reduced wasted effort by narrowing recruitment to only those potential participants known to meet initial BBH eligibility criteria. Potentially eligible Atlanta WIHS participants were notified of the BBH sub-study during their WIHS study visit and, if interested in study participation, were scheduled for a BBH screening visit during which final eligibility was assessed and informed consent obtained.

During the COVID-19 pandemic, there was an 11-month institutional research pause at Emory University, during which time WIHS transitioned to MWCSS. No recruitment occurred during the pause. Once research activities resumed, the efficacy of prescreening using information previously collected in WIHS became limited as it was no longer current enough for BBH pre-screening purposes and new MWCSS pre-screening information was not yet available. BBH adapted to these changes by redoubling recruitment efforts to make up for increasing screen-fails.

### Data collection

To streamline data collection, minimize costs, and reduce participant burden, BBH utilizes as much data as possible collected by the parent study: social and demographic characteristics, medication use and adherence, health behaviors, health history, blood chemistry and lipid panels. New BBH data collection is limited to outcomes assessments, important Project-specific covariates, and specific medical history questions.

Entry, one- and two- year follow-up visits were scheduled to take place within 6 months of a corresponding semiannual WIHS visit in order to minimize the time delay between parent study data collection (WIHS visit) and the sub-study data collection (BBH visit). After the MWCCS transition, the parent protocol changed to only require one annual visit from participants. Consequently, MWCCS data are now potentially widely separated in time (up to one year) to the corresponding BBH visit. In response, BBH investigators reevaluated the chronological windows for optimal data collection and the protocol was revised to ensure adequate timeliness of previously designated parent-study data elements.

**Fig 1:**
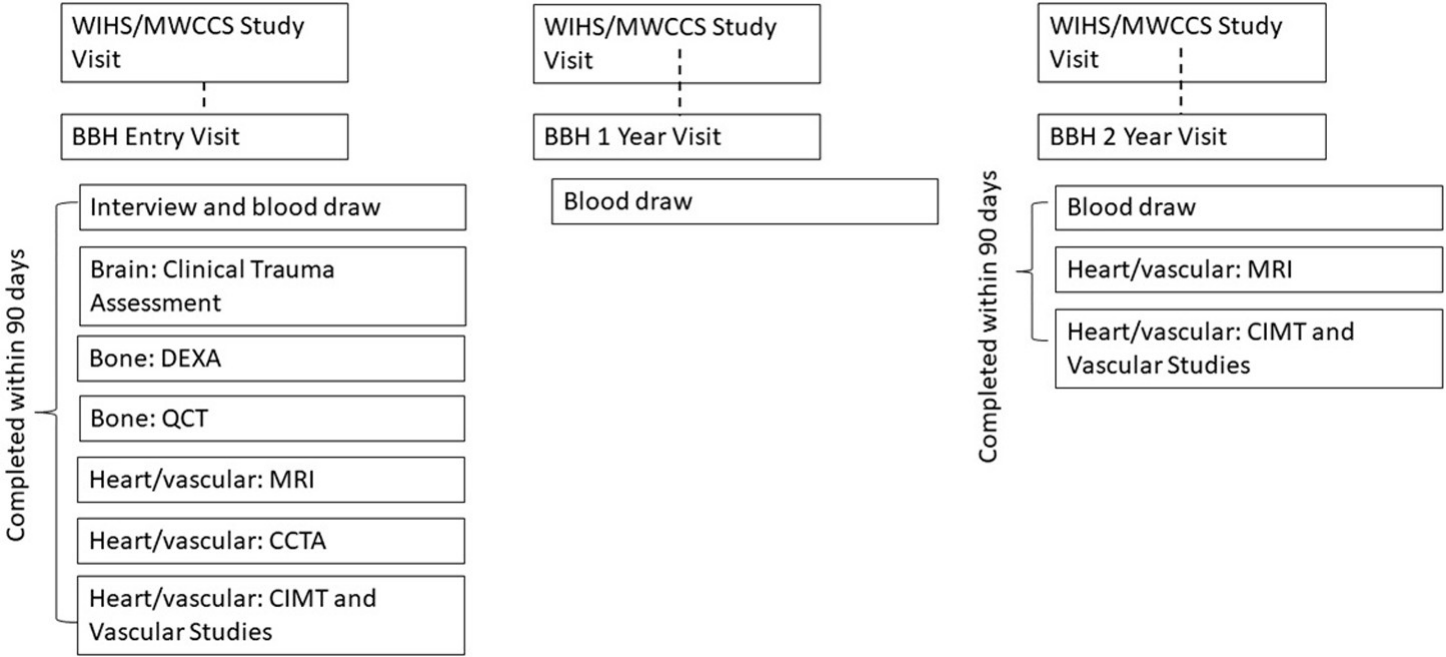
Brain, Bone, Heart (BBH) study visit components and schedule. BBH: Brain, Bone, Heart Study; MWCCS: Multicenter AIDS Cohort Study (MACS)/Women’s lnteragency HIV Study (WIHS) Combined Cohort Study (MWCCS); DEXA: dual energy X-ray absorptiometry; QCT: quantitative computed tomography; MRI: magnetic resonance imaging; CCTA: coronary computed tomography angiography; CIMT: carotid intima-media thickness.

BBH collects Project-specific data at study entry, one- and two-years post-study entry (see Fig 1). The entry visit includes an interview, selected bloodwork, and collection of specimens, carotid MRI, coronary computed tomography (CT) angiography, carotid intima-media thickness test (CIMT), vascular studies, quantitative computed tomography (QCT), dual energy X-ray absorptiometry (DEXA), and clinical assessment of trauma. The one-year visit includes collection of specimens only. The two-year visit includes specimen collection, CIMT, vascular studies, and carotid MRI.

Due to the complexity of participating in three separate but linked research Projects across multiple physical locations lasting variable lengths of time, BBH protocols require participants to complete all necessary Project components for each time point within 90 days after completion of the first component. Information about BBH data collection is summarized in Table 2. S1 Appendix describes data collection methods. BBH participants were compensated for each component of the study they chose to participate in.

**Table 2:** Description of information collected in Brain, Bone, Heart (BBH) study.

Category	Description
Health and Physical exam	Blood pressure, height, weight, BMI, fracture history, personal and family cardiovascular history
Blood testing	Blood count, chemistry panel, liver panel, fasting lipid profile, serum Vitamin D, parathyroid hormone
HIV-related	HIV-1-RNA PCR, CD4 T-cell counts
Inflammatory cytokines	hsCRP, TNFα, IL-6, IL-1β
Hormones and hormone receptors	Estrogen receptor gene expression (ERα, Erβ), AMH, FSH, estradiol
Structured Clinical Interview	Trauma exposure: Trauma Events Inventory, Childhood Trauma Questionnaire; PTSD: Clinical Administered PTSD Scale; Psychiatric Diagnosis: Mini International Neuropsychiatric Interview
Skin conductance	Psychophysiological Assessment of Hyperarousal
Serum markers of bone resorption	CTx, TRAP5b
Serum markers of bone formation	Osteocalcin, PINP
Bone mineral density	QCT and DEXA of lumbar spine, total hip, femur neck
Osteoclastogenic Factors	OPG, RANKL (total, T cell, B cell, monocyte)
Circulating progenitor cells	Absolute counts of target cell subsets and absolute mononuclear cell count: mononuclear cells (CD45dim population) expressing CD34+, CD133+, VEGF2R+, and CXCR4 epitopes either singly or in combination
Arterial stiffness	Pulse wave velocity, radial pulse wave analysis
Vascular profile	Brachial artery FMD
Coronary CT angiography	CCTA: Coronary plaque characteristics, Society of Cardiovascular
	Computed Tomography 5-point scale of obstructive stenosis [45], presence of atherosclerosis, number of vessels with ≥ 50% stenosis, Duke CAD prognostic index [46]
Carotid arterial disease using MRI/Ultrasound	MRI: vessel wall area, mean wall thickness averaged over circumferential locations, presence of plaque, plaque characteristics
	CIMT (ultrasound): Carotid intima-media thickness, the distance between the junction of the lumen and intima and that of the media and adventitia

BMI: body mass index; hsCRP: high sensitivity C-reactive protein; TNFα: tumor necrosis factor-α; IL-6/1β: interleukin-6/1β; ER: estrogen receptor; AMH: anti-mullerian hormone; FSH: follicle stimulating hormone; PTSD: to posttraumatic stress disorders; DEXA: dual energy X-ray absorptiometry; QCT: quantitative computed tomography; CTx: C-terminal telopeptide; PINP: procollagen type I N-terminal propeptide; FMD: flow mediated dilation; CT: computed tomography; CCTA: coronary computed tomography angiography; CAD: coronary artery disease; MRI: magnetic resonance imaging; CIMT: carotid intima-media thickness.

### Scheduling and logistics

After enrolling BBH, every effort is made to schedule participants for data collection in a way that minimizes patient burden while maximizing data collected across Projects. Scheduling is based on a complex synthesis of a participant’s availability, their willingness to undergo more than one procedure in a day, the time needed to travel to the fixed location of scanning equipment, time needed to complete each procedure, and equipment/personnel availability. As a result, a given participant may have from 1 to 7 separate scheduled appointments to complete their entire entry visit. To facilitate this, BBH staff developed and implemented a database that is used for scheduling and tracking to ensure that each participant completes all study components in the right order within each Project-specific time window over the course of BBH (S2 Appendix describes BBH databases).

To mitigate loss to follow up, during enrollment participants receive an information packet that describes each Project’s data collection process, the location of each procedure, and contact information for a coordinator who can help if transportation issues arise.

Information on participant safety and quality control is in S2 Appendix. STROBE [47] checklist information is in S2 Table.

### Contingencies

A challenge to BBH recruitment is the simultaneous recruitment of participants into the Atlanta MWCCS site, which uses the same clinical location and has overlapping staff. Although designed to be a sub-study, in order to meet recruitment goals, starting in February 2022 BBH is recruiting participants outside of or not yet enrolled into MWCCS. Non-MWCCS BBH participants are recruited from the same clinic locations as the MWCCS participants and complete a longer entry interview that additionally collects the subset of information not available from MWCCS for them. Non-MWCCS BBH participants participate in all BBH components as described in Fig 1. Another challenge is BBH participant refusal to participate in some Project components (example, MRI refusal due to claustrophobia). In response, the BBH has a contingency plan to enroll additional participants as needed for those components (and all linked components) into BBH so that all projects achieve the required sample size.

### COVID-19 mitigation

BBH recruitment was about one-half completed when research was institutionally-paused due to the COVID-19 pandemic. At the time of the research pause, some participants had only partially completed all components of the entry visit. The mitigation strategy allows for all missing entry visits components to be obtained in a new 90-day window along with another blood draw to ensure lab results concordant in time with the BBH outcomes data. Participants with entry visits >18 months from when research restarted were ineligible for their one-year visit. Although BBH staff make every effort to ensure research participant safety, a new challenge is encouraging participants to attend scheduled in-person visits during the COVID-19 pandemic.

## Discussion

BBH investigates a critical knowledge gap around examining the role of estrogen decline in women, on HIV pathology and its associated end organ damage through a complex, innovative, nested, tripartite study design. There are multiple benefits to combining three disparate Projects into a unified research enterprise, perhaps the greatest of which is the improvement in efficiencies, including cost, effort, and patient recruitment. A closely related benefit is that a tremendous amount of data is collected on one cohort of participants, providing an opportunity to characterize many different aspects of their health simultaneously. Another important factor is the synergy that occurs when researchers from different fields collaborate on a common goal, facilitating innovation and broadening the reach of novel ideas beyond their scientific field of origin. Inflammation and estrogen deficiency are common links between BBH Projects, providing an opportunity to study the underlying mechanisms that converge in the brain, bone, heart/vascular organ systems- an approach not feasible by any Project individually. As a result, BBH is well poised for these team science breakthroughs as it includes researchers from: infectious diseases, cardiology, psychiatry, hematology, neurobiology, biostatistics, psychology, behavioral science, and endocrinology.

The unique, nested, tripartite study design of BBH efficiently leverages limited resources and fosters team science discovery but is also organizationally complex and vulnerable to disruptions, such as the changes to the parent study and the global COVID-19 pandemic. BBH successfully adapted to these challenges due to strong study infrastructure and intra-organizational communication. Rigor and reproducibility in the methods described above were especially important to adapt to new parent study protocols, implement COVID-19 mitigation strategies, and to incorporate contingency plans to address known challenges. Strong communication at all levels has allowed BBH to function normally despite several ID clinician-scientist team members being diverted to provide care for COVID-19 patients and to participate in COVID-19 vaccine/therapeutic trials and other studies. Monthly meetings of BBH leadership and a separate monthly meeting of BBH leaders with the study investigators, coordinators, and data managers facilitates prompt recognition of issues as they arise, encourages thoughtful, inclusive discussion of potential solutions and their subsequent rapid deployment and evaluation.

The ultimate goal of BBH is to use HIV as a model to understand the impact of infectious diseases on the interaction between sex hormones and end organ disease. A greater understanding of the interplay between chronic HIV infection, age-related estrogen decline, chronic inflammation, and brain, bone, and heart health can point toward novel preventative and therapeutic options for WLH and open avenues for research into other age-related comorbidities associated with HIV. Further, this paradigm can serve as a model for other infectious diseases research. For example, women tend to have lower COVID-19 mortality and disease severity than men [48], and estrogen levels are associated with lower disease severity and inflammatory markers [49]. The BBH study design could be leveraged to determine mechanisms by which sex hormones influence COVID-19 outcomes across a range of organ systems. This unique study design is well-suited to the increasingly complex understanding of infectious disease outcomes.

## Conclusion

BBH’s research focus is on the HIV-host pathogen interaction as a model for probing the influence of SABV on the pathology and pathogenesis of infectious diseases. The Emory SCORE is addressing their research mission of identifying the role of biological sex differences on the health of women through the conduct of a unified, tripartite longitudinal research study, with each Project led by teams of investigators who have diverse and complementary training and skills. BBH is poised to provide insight into sex and HIV associations with the neuro-hypothalamic-pituitary-adrenal axis, skeletal, and cardiovascular systems despite several major, unexpected challenges.

## Supporting information

S1 Appendix List of methods used in Brain, Bone, Heart (BBH) Study. (DOCX)

S2 Appendix Additional Brain, Bone, Heart (BBH) information. (DOCX)

S3 Appendix Additional protocol information for animal subjects. (DOCX)

S1 Table Power calculations for each specific aim of the Brain, Bone, Heart (BBH) study projects. (DOCX)

S2 Table Brain, Bone, Heart (BBH) study STROBE statement checklist. (DOCX)
