# Supplementary material for: Bone, Brain, Heart study protocol: A resilient nested, tripartite prospective cohort study of the role of estrogen depletion on HIV pathology
Source: PLoS One. 2022 Aug 3;17(8):e0272608. doi: 10.1371/journal.pone.0272608 (PMC9348736; doi:10.1371/journal.pone.0272608)
Supplement: S1 Appendix — (DOCX) [file pone.0272608.s001.docx]

Supplement for Bone, Brain, Heart Study protocol: A resilient nested, tripartite prospective cohort study of the role of estrogen depletion on HIV pathology

Appendix A: List of methods used in Brain, Bone, Heart (BBH) Study

# Study entry

The following tests will be performed on a fee for service basis in a Clinical Laboratory Improvement Amendments (CLIA) certified laboratory:

## Covariates/potential confounders

Pregnancy*

Urine pregnancy test will be performed for participants with childbearing potential.

Specimen collection

50 mL of peripheral blood harvested for serum, plasma, and PBMC. Plasma and serum will be stored at -80C until analyzed. Residual PBMC will be cryopreserved for future use.

Blood testing*

Blood testing will include complete blood count, chemistry-14 panel, fasting lipid profile, pregnancy test (for women of childbearing age), HIV-1-RNA PCR and CD4 T-cell counts.

### Urinalysis*

Urine is collected for complete analysis.

Serum vitamin D and parathyroid hormone intact

Serum vitamin D and Parathyroid hormone intact will also be performed.

## Exposure variables

HIV testing*

Negative HIV sero-status will be confirmed in healthy volunteers by rapid ELISA test after pre-test counseling. In persons living with HIV, definitive proof of HIV diagnosis by ELISA test, western blot and/or plasma HIV-RNA PCR will be documented.

* From the immediately preceding Women’s Interagency HIV Study (WIHS) or Multicenter AIDS Cohort Study (MACS)/Women’s Interagency HIV Study Combined Cohort Study (MWCCS) visit within previous year

# Project 1: Brain

## **Outcomes, exposures, potential confounders/covariates^†^**

Hormones

Estradiol, FSH, AMH will be measured using mass spectrometry[1] with a triple quadrupole mass spectrometer (AB Sciex 6500+ ) coupled to a Shimadzu Nexera UPLC.

Inflammatory cytokines

Serum concentrations of IL-6, TNF-α, and IL-1β will be measured with AlphaLISA Detection Kits from Perkin Elmer.

Estrogen receptor (ER) α and β gene expression

Samples will be assessed using qRT-PCR. RNA will be isolated from PBMCs and then assessed using TaqMan Gene Expression Assays. We will use a system from Applied Biosystems (TaqMan® PreAmp Master Mix Kit) that provides a rapid, simple, and sensitive method for preamplifying small amounts of cDNA without introducing amplification bias.

## Exposures

Estrogen receptor response to estradiol

We will assess the response of ERs in culture by stimulating with 17β-estradiol and then using Estrogen Receptor Signaling RT2 Profiler PCR Arrays (Qiagen) to assess 84 ER-regulated genes to begin to develop a picture of ER function in women living with HIV (WLH) with the highest and lowest inflammatory signals within our study. PBMCs will be suspended in RPMI-1640 media with 10% stripped FBS and supplements, and 10^6 cells per well will be incubated with 17β-estradiol (0.5 ng/ml or 30 ng/ml132) in sterile 24-well culture plates in a humidified atmosphere at 37°C in 5% CO2 for 2 hr. After incubation, the samples will be centrifuged and supernatant collected and stored at -80°C until batch assessment via PCR array.

Estrogen receptor response to inflammatory stimulus

We will assess the efficacy of ERs in response to an ex vivo inflammatory challenge. PBMC will be suspended in RPMI-1640 media with 10% FBS and supplements, and 10^6^ cells per well will be incubated with LPS (0 ng/ml or 30 ng/ml) in sterile 24-well culture plates in a humidified atmosphere at 37°C in 5% CO2 for 6 hr. After incubation, the samples will be centrifuged and supernatant collected and stored at -80°C until batch assaying of cytokines as control conditions for following experiment. RNA will be isolated and ER subtype expression assessed as above to determine if LPS stimulation alters ERs. Assessment of ERs in the 0 ng/ml LPS condition will be evaluated for predictive potential of cytokine outcomes.

Estrogen receptor subtype modulation of inflammatory stimulus

In order to determine relative contributions of ERα and ERβ to anti-inflammatory actions, PBMCs will be cultured with both ER subtype-specific agonists and LPS. PBMCs will be suspended in RPMI-1640 media with 10% FBS and supplements, and 10^6^ cells per well will be incubated with either control, estradiol, an ERα agonist, and ERβ agonist, or dexamethasone. Estradiol will be stimulated through application of 17β-estradiol and is planned to be applied at 30 ng/ml, but results of the ER response to estradiol may alter this concentration. The ERα agonist PPT (4,4’,4”-(4-propyl-[1H]-pyrazole-1,3,5-triyl)*tris*-phenol; 0.1pg/ml) will be used to assess the effect of ERα. PPT has a 410-fold greater affinity for ERα over ERβ at the defined concentration and should be specific to ERα[2]. The ERβ agonist DPN (2,3-*bis*(4-hydroxyphenyl)-propionitrile; 10 pg/ml) will be used to assess the effect of ERβ). DPN has a 70-fold greater affinity for ERβ over ERα at this concentration[3] and should be specific to ERβ. As a positive control, dexamethasone, a synthetic glucocorticoid receptor agonist (DEX; 10-5 mol/liter) will be applied to cultures. All culture additions outlined will occur in sterile 24-well culture plates in a humidified atmosphere at 37°C in 5% CO2 for 2 hr prior to LPS. Cultures will then be incubated with lipopolysaccharide (LPS; 0 ng/ml or 30 ng/ml) for 6 hr. After incubation, the samples will be centrifuged and supernatant collected and stored at -80°C until batch assaying of cytokines and pellet will be retained for further analysis if needed for follow up studies.

Clinical assessment of trauma exposure and Post-Traumatic Stress Disorder (PTSD)

Participants will be interviewed by a trained clinician on the following psychological assessment instruments:

- Traumatic Events Inventory (TEI): The TEI assesses lifetime experience and frequency of fourteen types of traumatic events[4, 5].
- Childhood Trauma Questionnaire (CTQ): The CTQ is a 28-item psychometrically validated, self-report inventory assessing self-reported level of child abuse and neglect. This instrument yields categorical and continuous measures of abuse and neglect[6].
- Clinician Administered PTSD Scale (CAPS): The CAPS is a reliable, valid interview measure for DSM-5 PTSD that yields PTSD symptom frequency and severity. The CAPS data will be used create continuous variables reflecting the combine frequency and severity of intrusion, avoidance, and hyperarousal, as well as a diagnosis of PTSD. The CAPS also assesses duration of PTSD symptoms[7].
- Mini International Neuropsychiatric Interview (MINI): The MINI is a semi-structured interview designed to diagnose Axis I pathology. We will use the MINI to assess co-occurring conditions using the current and lifetime depression, anxiety, schizophrenia and bipolar spectrum disorders and substance dependence disorders[8].

Psychophysiological assessment of hyperarousal

Skin conductance (SC) data will be acquired using the eSense (Mindfield Biosystems) system connected to an iPad. Two 5 mm Ag/AgCl electrodes filled with isotonic paste will be attached to middle phalanges of the second and fourth finger of the non-dominant hand during administration of a trauma interview. The procedure will last 15 minutes. After collecting baseline SC data, the Standardized Trauma Interview (STI) will be administered while SC level is continuously measured[9].

^†^ Classification as outcome, exposure, potential confounder/covariate depends on BBH Project and Specific Aim.

# Project 2: Bone- Human subjects

## **Outcomes**

Cell and cytokine quantification

PBMC will be analyzed by FACS and lymphocyte T cell, B cell, and macrophage/monocyte specific production of OPG, RANKL and TNF-α will be quantified on a per cell basis using flow cytometry.

Circulating cytokine quantification

Total circulating levels of osteoclastogenic regulators (RANKL, OPG and TNF-α) in plasma/serum will be measured by ELISA.

Markers of bone turnover and formation

Bone turnover, quantified by serum markers of bone resorption (CTx and TRAP5b) and formation (osteocalcin and PINP).

Dual-energy X-ray absorptiometry (DEXA) scan

Bone mineral density (BMD) in the lumbar spine, total hip, and hip/femur neck will be quantified by DEXA using a human lunar bone densitometer.

Enzyme-linked immunosorbent assays (ELISA)

ELISA from Immunodiagnostic Systems, (Scottsdale, AZ) will be used according to the manufacturer’s instructions to quantify serum CTx and TRAP5b (resorption markers); OCN and P1NP (formation markers)[10, 11] and OPG, RANKL, and TNFα as previously published by our group[12-14]. Note that one central laboratory facility (the clinical and translational research lab Directed by Dr. Ofotokun) is used to measure all biomarkers including CTx and OCN to minimize assay variability and eliminate lab to lab variability. These markers are stable and can be reliably quantified in stored serum.

Fluorescence activated cell sorting (FACS)

Approximately 50 mL of peripheral blood will be harvested for serum, plasma, and PBMC. Plasma and serum will be stored at -80^°^C and PBMC will be cryopreserved in liquid nitrogen until analyzed. PBMC will be analyzed by FACS and lymphocytes (B- and T-cells) stained for OPG, RANKL and TNFα.

Skeletal imaging – lumbar spine, total hip and femoral neck

BMD will be assessed using Lunar prodigy scanner (GE Lunar, Madison, WI) DXA machine and Encore Software, v.2010 13.31. Osteopenia and osteoporosis per WHO criteria[15]. Skeletal pQCT - segmented to generate separate cortical and trabecular bone BMD at femur neck and lumbar spine as previously described[16].

# Project 2: Bone- Mouse model

Animal housing and husbandry

Female wildtype (WT) and TCRβ knockout (KO) mice on the C57BL6/J background (The Jackson Laboratory, Bar Harbor, ME) are housed under specific-pathogen-free conditions and fed γ-irradiated low phytoestrogen PicoLab Select 5V5R mouse chow (LabDiet, St. Louis, MO) and autoclaved water ad libitum. The temperature of the animal facility is kept at 23°C (±1°C) with 50% relative humidity and a 12:12-hour light/dark cycle.

Mice ovariectomy

The hair surrounding the surgical site is removed with electric clippers or depilatory cream (Nair), and the skin cleaned by a minimum of three alternating applications each of isopropyl alcohol and betadine antiseptic. Ocular ointment is placed on the eyes to prevent exposure keratitis. Instruments are sterilized by initial autoclaving and maintained sterile between procedures by means of a bead sterilizer. The mice are anesthetized using 3% isoflurane (in 100% oxygen), which is maintained throughout surgery utilizing a vaporizer. Anesthesia waist gas is scavenged by means of a charcoal filter. Mice are tested for lack of response to pedal reflex prior to surgery and the operation performed during the “surgical plane of anesthesia.” The mouse is placed ventral side up and a 1 cm incision made in the skin in the middle of the abdomen below the rib cage (anterior side of the body). The left ovary is exposed, a suture placed adjacent to the ovary on the fallopian tube, and the ovary removed with surgical scissors or an electrocautery device. This procedure is repeated for the right ovary. The muscle layer and the skin are subsequently closed with interrupted monofilament (4-0) sutures. For sham ovariectomy, the procedure is performed exactly as described above for ovariectomy, however, the ovary is exposed without removal and is then replaced into the abdominal cavity, and the muscle layer and skin closed with interrupted monofilament (4-0) sutures. After surgery, animals are returned to their cages, which are partly placed on a heating pad for thermal support to avoid hypothermia until they are awake and ambulatory. Analgesia is provided immediately before surgery, by an intraperitoneal injection of Meloxicam (5mg/Kg) with follow-up doses given 24- and 48-hours after surgery. External sutures are removed after 10-14 days when the surgical incision has healed.

17𝛽-estradiol pellet insertion

For mice receiving estrogen replacement, a 17𝛽-estradiol pellet is implanted subcutaneously at the back of the neck, immediately following ovariectomy while the mouse is still anesthetized from ovariectomy surgery, and before recovery. The mouse is placed dorsal side up, and hair removed from the surgical site with electric clippers or depilatory cream (Nair) and the area thoroughly cleaned 3 times with alternating alcohol and betadine solutions. A 0.5 cm incision is made in the skin on the lateral side of the neck and a small subcutaneous pocket formed through the incision with forceps. One 0.001mg, 60 days release, 17𝛽-estradiol pellet (Innovative Research of America, Sarasota, FL) is implanted into the pocket. The skin is then closed with interrupted monofilament sutures (size 4-0). Sutures are removed after 10-14 days when the surgical incision has healed.

T cell adoptive transfer

CD3+ T cells (>97% pure) are transplanted into TCRβ KO mice lacking αβ T cells by adoptive transfer of 1 X 10E4 to 1 X 10E5 CD3+ T cells by lateral tail vein injection, or by retro-orbital injection. T cells for transplant are immunomagnetically purified from single-cell suspensions of disaggregated spleens from C57BL6 female mice post sacrifice by negative selection, performed under aseptic conditions, using the EasySep Mouse T Cell Isolation Kit (Stemcell. Vancouver, BC).

## **Outcomes**

Dual-energy X-ray absorptiometry for bone densitometry in mice

Prospective bone densitometry to quantify bone mineral density (BMD) is performed in mice using dual-energy X-ray absorptiometry (DXA). BMD in mice is quantified at baseline (0-time) and additional 2- to 4-week intervals for the duration of the experiment. DXA is performed under general anesthesia (Isoflurane 1.5- 2.5% in 100% oxygen using a vaporizer) using either a PIXImus 2 bone densitometer and GE Software (GE Medical Systems) or a Kubtec Parameter 2D Cabinet X-ray System with Digimus analysis Software (Kubtec, Stratford, CT). Only one machine is used for the duration of the study. Both devices are designed specifically for rodents. Whole-body DXA is performed, and region of interest boxes placed to determine BMD for femurs and spine.

Micro–computed tomography

Micro-computed tomography (μCT) is performed in the L3 vertebral body and the mid-femoral diaphysis (cortical bone) and distal metaphysis (trabecular bone) of the right femur ex vivo, to assess cortical and trabecular bone volume and microarchitecture. Mice are euthanized, and bones isolated by dissection, followed by soft-tissue removal. Bones are fixed and stored in 70% ethanol at 4oC until analyzed on a microCT 40 scanner (Scanco Medical, Brüttisellen, Switzerland). The scanner is calibrated weekly with a factory-supplied phantom. Tomographic slices are taken using isotropic 6-μm voxels, with an integration time of 200 ms. The tube voltage and current are 70 kVp and 114 mA, respectively. Trabecular bone is segmented from the cortical shell, beginning approximately 0.5 mm from the distal growth plate of the femur. Projection images are reconstructed using the auto-contour function for trabecular bone. Femoral cortical bone is quantified at the mid-diaphysis from 100 tomographic slices spanning 0.6 mm. Representative vertebral samples based on the mean ratio of the bone volume to the total volume are reconstructed to generate 3-dimensional visual representations. Thresholding is achieved by visual inspection and comparison of preview and slice-wise grayscale 2D images. Once established, the same threshold value is used for all measurements. Indices and units are standardized per published guidelines for the assessment of bone microstructure in rodents using micro-computed tomography[17].

# Project 3: Heart/vascular

## Outcomes

CCTA image acquisition

Patients will not be considered for study inclusion if they had contraindications to CT or to the administration of iodinated contrast medium. CCTA image acquisition will be performed on a 3rd-generation DSCT system (SOMATOM Force, Siemens Healthcare, Forchheim, Germany) in accordance with Society of Cardiovascular Computed Tomography (SCCT) guidelines[18, 19]. To bring the heart rate <70 bpm in sinus rhythm, intravenous metoprolol may be used. Sublingual nitroglycerine will be administered. Patients will undergo non-contrast enhanced coronary artery calcium score (CACS) acquisition, which is followed by a contrast-enhanced CCTA. For CCTA acquisition, a prospectively ECG triggered adaptive sequential-mode acquisition protocol will be applied and images will be acquired at full nominal tube current between 30% and 80% of the RR interval, depending on the heart rate. The acquired scan length will range from the carina to the cardiac apex. Automated tube current modulation (CARE Dose4D, Siemens) and automated tube voltage selection (ATVS, CARE kV, Siemens) with available tube voltage settings ranging from 70–130 kV in 10-kV increments will be used. Further scan parameters will be as follows: adaptive detector collimation varying from 96–192 in steps of 8 x 0.6 mm, gantry rotation 0.25 s, pitch 1.0, matrix size 512 x 512 pixels. Vessel attenuation will be achieved with the use of intravenously administered iodinated contrast agent at a flow rate adapted to the tube voltage followed by a 50-mL saline chaser injected at the same flow rate. Contrast medium volume will be adapted to the tube voltage ranging from 50 to 80 mL at a flow rate of 4-5 mL/s. A region of interest (ROI) was placed within the descending aorta at the level of the carina, with the scan being automatically triggered 4 seconds after a threshold of 100 Hounsfield Units (HU) will be reached. All scan lengths will range from the carina to the cardiac apex. Dose reduction strategies will be employed whenever feasible[20]. DICOM images will be stored.

CCTA image analysis

Blinded core lab readers will perform CCTA interpretation after adjusting thresholds as required[21]. Datasets were transferred to a dedicated post-processing workstation (*syngo*.via VB10B, Siemens Healthineers, Forchheim, Germany). All CCTA images were assessed for image quality on a 4-point Likert scale: 1 = non-diagnostic, impaired image quality limited by excessive image noise and/or poor vessel wall delineation; 2 = adequate, reduced image quality with excessive image noise and poor vessel wall delineation and limitations in contrast remain evident; 3 = good, impact of poor image noise, vessel wall delineation and contrast condition are minimal; 4 = excellent, image noise not perceivable, optimal vessel wall delineation and excellent vessel lumen enhancement. Angiographic Coronary Artery Disease Extent and Severity will be measured using the SCCT 5-point scale, all angiograms will be scored as 0%; 1-24%; 25-49%, 50-69%, 70-99%, and 100% according to the CAD-RADS score[22, 23]. Plaque will be defined as any tissue >1mm^2^ within or adjacent to the lumen that can be identified in >2 planes[24]. The number of vessels with ≥50% stenosis will be counted and the Duke CAD prognostic index will be calculated[25].

CCTA high risk plaque (HRP) features examined

(1) Lowest Attenuation Plaque (LAP): Multiple regions-of-interest (ROI) will be placed on a cross-sectional orthogonal projection of the plaque. The ROIs will be placed in >3 sites with the lowest HU density, with a HU <30 signifying LAP[26-28]. Normalization will be performed for body mass index, peak iodinated contrast attenuation, and signal-to-noise. (2) Remodeling: Arterial remodeling will be defined as the diameter at a site with plaque compared to a reference segment that is from the most proximal normal appearing arterial lumen. The remodeling index (RI) will be calculated as maximal lesion diameter/reference diameter and categorized as: 1) expansive (RI>1.1); intermediate (RI 0.9-1.1), and negative (RI <0.9)[29]. (3) Plaque Composition: Plaque composition will be classified as calcified for greater and non-calcified for lesser attenuation than the contrast-enhanced lumen, while admixtures will be rated by volumetric proportion on a 5-level scale: (a) non-calcified (0% calcified plaque [CP]), (b) mostly non-calcified (1-30% CP), (c) mixed (30-70% CP), (d) mostly calcified (70-99% CP), and (e) calcified (100% CP)[30, 31]. Plaque composition scores will be calculated by summing the number of segments by plaque type[32]. (4) Plaque Burden (PB): PB will be reported by volume, in order to maximize cross-study comparisons[21, 33-35]. By curved MPRs, cross-sections of 1 mm increments of the artery will be examined. In each cross-section, we will (a) trace the outer artery contour (= both the plaque *and* lumen area), (b) trace the contrast-enhanced lumen (= lumen area alone), and (c) subtract these two areas (= plaque area alone). Plaque volume for each cross-section will be calculated by multiplying area by the increment length (i.e., 1 mm), with the sum of these volumes equaling total plaque volume. We will perform analyses for PB by composition, with CP measured on each cross-section, and the difference between the total plaque area and CP area defining the non-CP area. (5) Minimal Luminal Area (MLA): MLA will be obtained by identifying all segments with plaque for the point of minimum area within an abnormal segment using curved MPRs and cross-sections[36], with the most proximal normal coronary segment serving as reference.

Carotid MRI image acquisition

Carotid MRI will be performed on a 3T MRI system (Siemens Prisma, Siemens Medical Solutions, Malvern, PA) using a multi-channel phased-array coil. A gradient-echo, 3-plane localizer will be performed to locate carotid artery bifurcations, and 3D, time-of-flight (TOF), non-contrast angiographic images will be acquired in the transverse orientation with an in-plane resolution of 0.7 mm and a slice thickness of 1.0 mm. Anatomic landmarks on the coronal and sagittal maximum intensity projection (MIP) images created from the TOF scan will be used to center subsequent imaging sequences on the top of left carotid bifurcation. T1 weighted (T1W), T2 weighted (T2W) imaging will be performed using a 2D, double inversion-recovery, black-blood fast spin echo sequence with in-plane resolution of 0.5 mm and a slice thickness of 2.0 mm[37]. In addition, a 3D multi-contrast STAR MATCH sequence of the carotid image will be acquired in the coronal plane[38]. The 2D images will be used for determining wall thickness in the common carotid and the carotid bulb, and the 3D multi-contrast images will be used for plaque characterization and overall plaque burden in the cerebral vasculature.

Carotid MRI image analysis

Image analysis will be performed using a dedicated vessel analysis package (VesselMASS, LUMC, Leiden, Netherlands) that displays the entire stack of images, and has a work window which can magnified for accurate tracing of vessel boundaries in any single slice. An investigator blinded to the clinical information will manually trace the outer vessel boundary (defined as the vessel wall-soft tissue interface) and the inner vessel boundary (defined as the vessel lumen-wall interface) in the left and right carotid of each patient. Images from all time points will be reviewed together to ensure alignment of carotid slices between baseline and each follow-up time point. Measurements will be made on images acquired in the common carotid arteries and separately in the bulb slices and will include: (1) vessel wall area defined as the difference between the outer vessel wall area and the lumen area; (2) mean wall thickness averaged over circumferential locations (WT). Each cross-sectional image will be divided into six contiguous sectors and the mean WT calculated automatically as the mean of all individual sectors[39, 40]. (3) Presence of plaque is defined as wall area with WT >2 mm[41]. (4) Plaque characterization analysis will be performed on a pixel-by-pixel basis using histology-validated criteria; the different signal intensities that result from the use of multi-contrast weighted imaging will be used to identify plaque constituents[42]. The volume of each plaque constituent will be determined for each subject at each time point.

CIMT and carotid plaque

Carotid intima-media thickness using ultrasound: Carotid IMT, the distance between the junction of the lumen and intima and that of the media and adventitia, is measured by means of B-mode ultrasound of the carotid arteries following a standardized methodology. Early atherosclerosis in superficial arteries can be assessed noninvasively by B-mode ultrasound. Even in the absence of discrete plaque or stenosis, the combined thickness of the arterial intima and media can be measured with considerable precision by this technique[43]. Increased CIMT is associated with prevalent CVD and risk of future MI and stroke[43-45].

## Potential confounders/covariates

Vascular function testing

Vascular tests be performed following an overnight fast. After a 20-minute rest in a quiet, temperature-controlled room, resting blood pressure was measured.

Arterial stiffness

We will measure augmentation index (AIX) and carotid-femoral pulse wave velocity (PWV) for assessment of arterial wave reflections and stiffness, respectively, derived using the Sphygmocor device (Atcor Medical, Australia) as previously described[46]. Briefly, high- fidelity sequential pressure waveforms are from the radial artery using a tonometer. Using a transfer function, central (aortic) pressure and the degree of pressure augmentation secondary to reflected waves from the periphery is estimated. AIX is then derived as augmented pressure/total central pulse pressure and is considered a composite marker of wave reflections and arterial stiffening. Due to its sensitivity to heart rate, a standardized value to 75 bpm is calculated and used for AIX for the purpose of this study. PWV will be determined by acquiring waveforms at the carotid and femoral arterial sites using EKG gating. Velocity [distance/time in m/s] was calculated by measuring the time interval between EKG R-waves and the recorded waveforms at each site, while distance between sites was measured manually. Quality control indices were evaluated at the time of study and non-acceptable readings discarded and tests were repeated. Reproducibility studies in our laboratory on 9 subjects on consecutive days have demonstrated a coefficient of variation of 3.8%, 20.3%, and 13.8% for PWV, AIX, and SEVR respectively.

Brachial artery flow mediated dilatation (FMD)

Endothelium-dependent brachial artery FMD will be measured to evaluate endothelium-dependent vasodilation as previously described[46]. Briefly, sonographic images showing a clear intima-media/adventitia interface, in addition to pulse Doppler velocities are obtained before and after suprasystolic cuff occlusion at the forearm. FMD is calculated as the percent dilation observed at one minute following cuff release. In our laboratory, the mean difference in FMD between assessments performed in 11 subjects on consecutive days was 1.26±0.76%, with a correlation coefficient of 0.75. The mean difference in the FMD between 2 readings of the same 11 measurements was 0.82±0.48% (r=0.97).

## Exposure

Circulating Progenitor Cell (PC) assays

300ul of venous blood will be incubated with 15ul FITC-CD34 (BD Biosciences), 15ul PE-VEGF-R2 (R&D system), 15ul PerCP-CD45 (BD Biosciences) 10ul APC-CD133 (Miltenyi), and 7ul of PE-Cy7-conjugated anti-CXCR4 (EBioscience, clone 12G5)[47-50]. Ammonium chloride is added to lyse red blood cells and then a staining medium (PBS with 3% heat-inactivated serum and 0.1% sodium azide) to stop lysis. After mixing, centrifugation and washing with PBS, 100ul of Perfect Count Beads (Caltag) (Invitrogen) are added to act as an internal standard for direct estimation of the concentration of target cell subsets. At least 1 million events will be acquired on a flow cytometer.  Absolute counts of target cell subsets will be determined together with absolute mononuclear cell count. PCs enumerated will include mononuclear cells (CD45^dim^ population) expressing CD34+, CD133+, VEGF2R+, and CXCR4 epitopes either singly or in combination.

# References

1. Field HP. Tandem mass spectrometry in hormone measurement. Methods Mol Biol. 2013;1065:45-74. doi: 10.1007/978-1-62703-616-0_4. PubMed PMID: 23996357.

2. Stauffer SR, Coletta CJ, Tedesco R, Nishiguchi G, Carlson K, Sun J, et al. Pyrazole ligands: structure-affinity/activity relationships and estrogen receptor-alpha-selective agonists. J Med Chem. 2000;43(26):4934-47. PubMed PMID: 11150164.

3. Meyers MJ, Sun J, Carlson KE, Marriner GA, Katzenellenbogen BS, Katzenellenbogen JA. Estrogen receptor-beta potency-selective ligands: structure-activity relationship studies of diarylpropionitriles and their acetylene and polar analogues. J Med Chem. 2001;44(24):4230-51. PubMed PMID: 11708925.

4. Binder EB, Bradley RG, Liu W, Epstein MP, Deveau TC, Mercer KB, et al. Association of FKBP5 polymorphisms and childhood abuse with risk of posttraumatic stress disorder symptoms in adults. JAMA. 2008;299(11):1291-305. Epub 2008/03/20. doi: 10.1001/jama.299.11.1291. PubMed PMID: 18349090; PubMed Central PMCID: PMCPMC2441757.

5. Schwartz AC, Bradley RL, Sexton M, Sherry A, Ressler KJ. Posttraumatic stress disorder among African Americans in an inner city mental health clinic. Psychiatr Serv. 2005;56(2):212-5. Epub 2005/02/11. doi: 10.1176/appi.ps.56.2.212. PubMed PMID: 15703352.

6. Bernstein DP, Stein JA, Newcomb MD, Walker E, Pogge D, Ahluvalia T, et al. Development and validation of a brief screening version of the Childhood Trauma Questionnaire. Child Abuse Negl. 2003;27(2):169-90. Epub 2003/03/05. doi: 10.1016/s0145-2134(02)00541-0. PubMed PMID: 12615092.

7. Blake DD, Weathers FW, Nagy LM, Kaloupek DG, Gusman FD, Charney DS, et al. The development of a Clinician-Administered PTSD Scale. J Trauma Stress. 1995;8(1):75-90. Epub 1995/01/01. doi: 10.1007/bf02105408. PubMed PMID: 7712061.

8. Sheehan DV, Lecrubier Y, Sheehan KH, Amorim P, Janavs J, Weiller E, et al. The Mini-International Neuropsychiatric Interview (M.I.N.I.): the development and validation of a structured diagnostic psychiatric interview for DSM-IV and ICD-10. J Clin Psychiatry. 1998;59 Suppl 20:22-33;quiz 4-57. Epub 1999/01/09. PubMed PMID: 9881538.

9. Hinrichs R, Michopoulos V, Winters S, Rothbaum AO, Rothbaum BO, Ressler KJ, et al. Mobile assessment of heightened skin conductance in posttraumatic stress disorder. Depress Anxiety. 2017;34(6):502-7. Epub 2017/02/22. doi: 10.1002/da.22610. PubMed PMID: 28221710; PubMed Central PMCID: PMCPMC5466496.

10. Immunodiagnostic Systems Inc. C-terminal telopetide of Collagen Crosslaps ELISA [cited 2017 January 30]. Available from: http://www.idsplc.com/products/serum-crosslaps-ctx-i-elisa-2/.

11. Immunodiagnostic Systems Inc. Osteocalcin N-mid ELISA Fountain Hills, AZ [cited 2017 January 30]. Available from: http://www.antibodies-online.com/kit/368354/N-MID+Osteocalcin+ELISA+Kit/.

12. Ofotokun I, Titanji K, Lahiri CD, Vunnava A, Foster A, Sanford SE, et al. A Single-dose Zoledronic Acid Infusion Prevents Antiretroviral Therapy-induced Bone Loss in Treatment-naive HIV-infected Patients: A Phase IIb Trial. Clin Infect Dis. 2016;63(5):663-71. doi: 10.1093/cid/ciw331. PubMed PMID: 27193748; PubMed Central PMCID: PMCPMC4981757.

13. Ofotokun I, Titanji K, Vunnava A, Roser-Page S, Vikulina T, Villinger F, et al. Antiretroviral therapy induces a rapid increase in bone resorption that is positively associated with the magnitude of immune reconstitution in HIV infection. AIDS. 2016;30(3):405-14. doi: 10.1097/QAD.0000000000000918. PubMed PMID: 26760232; PubMed Central PMCID: PMCPMC4712729.

14. Titanji K, Vunnava A, Sheth A, Lennox J, Ofotokun I, Weitzmann MN. B Cell Dysregulation Promotes HIV-induced Bone Loss. Journal of Bone and Mineral Research. 2013;28. PubMed PMID: WOS:000332035803249.

15. Kanis JA. Diagnosis of osteoporosis and assessment of fracture risk. Lancet. 2002;359(9321):1929-36. doi: 10.1016/S0140-6736(02)08761-5. PubMed PMID: 12057569.

16. Yin MT, Shu A, Zhang CA, Boutroy S, McMahon DJ, Ferris DC, et al. Trabecular and cortical microarchitecture in postmenopausal HIV-infected women. Calcif Tissue Int. 2013;92(6):557-65. doi: 10.1007/s00223-013-9716-8. PubMed PMID: 23460340; PubMed Central PMCID: PMCPMC3656136.

17. Bouxsein ML, Boyd SK, Christiansen BA, Guldberg RE, Jepsen KJ, Muller R. Guidelines for assessment of bone microstructure in rodents using micro-computed tomography. J Bone Miner Res. 2010;25(7):1468-86. Epub 2010/06/10. doi: 10.1002/jbmr.141. PubMed PMID: 20533309.

18. Abbara S, Arbab-Zadeh A, Callister TQ, Desai MY, Mamuya W, Thomson L, et al. SCCT guidelines for performance of coronary computed tomographic angiography: a report of the Society of Cardiovascular Computed Tomography Guidelines Committee. J Cardiovasc Comput Tomogr. 2009;3(3):190-204. Epub 2009/05/05. doi: 10.1016/j.jcct.2009.03.004. PubMed PMID: 19409872.

19. Raff GL, Abidov A, Achenbach S, Berman DS, Boxt LM, Budoff MJ, et al. SCCT guidelines for the interpretation and reporting of coronary computed tomographic angiography. J Cardiovasc Comput Tomogr. 2009;3(2):122-36. Epub 2009/03/11. doi: 10.1016/j.jcct.2009.01.001. PubMed PMID: 19272853.

20. Halliburton SS, Abbara S, Chen MY, Gentry R, Mahesh M, Raff GL, et al. SCCT guidelines on radiation dose and dose-optimization strategies in cardiovascular CT. J Cardiovasc Comput Tomogr. 2011;5(4):198-224. Epub 2011/07/05. doi: 10.1016/j.jcct.2011.06.001. PubMed PMID: 21723512; PubMed Central PMCID: PMCPMC3391026.

21. Cheng VY, Nakazato R, Dey D, Gurudevan S, Tabak J, Budoff MJ, et al. Reproducibility of coronary artery plaque volume and composition quantification by 64-detector row coronary computed tomographic angiography: an intraobserver, interobserver, and interscan variability study. J Cardiovasc Comput Tomogr. 2009;3(5):312-20. Epub 2009/08/28. doi: 10.1016/j.jcct.2009.07.001. PubMed PMID: 19709947.

22. Cury RC, Abbara S, Achenbach S, Agatston A, Berman DS, Budoff MJ, et al. Coronary Artery Disease - Reporting and Data System (CAD-RADS): An Expert Consensus Document of SCCT, ACR and NASCI: Endorsed by the ACC. JACC Cardiovasc Imaging. 2016;9(9):1099-113. Epub 2016/09/10. doi: 10.1016/j.jcmg.2016.05.005. PubMed PMID: 27609151.

23. Cury RC, Abbara S, Achenbach S, Agatston A, Berman DS, Budoff MJ, et al. CAD-RADS(TM) Coronary Artery Disease - Reporting and Data System. An expert consensus document of the Society of Cardiovascular Computed Tomography (SCCT), the American College of Radiology (ACR) and the North American Society for Cardiovascular Imaging (NASCI). Endorsed by the American College of Cardiology. J Cardiovasc Comput Tomogr. 2016;10(4):269-81. Epub 2016/06/20. doi: 10.1016/j.jcct.2016.04.005. PubMed PMID: 27318587.

24. Budoff MJ, Dowe D, Jollis JG, Gitter M, Sutherland J, Halamert E, et al. Diagnostic performance of 64-multidetector row coronary computed tomographic angiography for evaluation of coronary artery stenosis in individuals without known coronary artery disease: results from the prospective multicenter ACCURACY (Assessment by Coronary Computed Tomographic Angiography of Individuals Undergoing Invasive Coronary Angiography) trial. J Am Coll Cardiol. 2008;52(21):1724-32. Epub 2008/11/15. doi: 10.1016/j.jacc.2008.07.031. PubMed PMID: 19007693.

25. Min JK, Shaw LJ, Devereux RB, Okin PM, Weinsaft JW, Russo DJ, et al. Prognostic value of multidetector coronary computed tomographic angiography for prediction of all-cause mortality. J Am Coll Cardiol. 2007;50(12):1161-70. Epub 2007/09/18. doi: 10.1016/j.jacc.2007.03.067. PubMed PMID: 17868808.

26. Iriart X, Brunot S, Coste P, Montaudon M, Dos-Santos P, Leroux L, et al. Early characterization of atherosclerotic coronary plaques with multidetector computed tomography in patients with acute coronary syndrome: a comparative study with intravascular ultrasound. European radiology. 2007;17(10):2581-8. doi: 10.1007/s00330-007-0665-3. PubMed PMID: 17549491.

27. Motoyama S, Kondo T, Sarai M, Sugiura A, Harigaya H, Sato T, et al. Multislice computed tomographic characteristics of coronary lesions in acute coronary syndromes. J Am Coll Cardiol. 2007;50(4):319-26. doi: 10.1016/j.jacc.2007.03.044. PubMed PMID: 17659199.

28. Motoyama S, Kondo T, Anno H, Sugiura A, Ito Y, Mori K, et al. Atherosclerotic plaque characterization by 0.5-mm-slice multislice computed tomographic imaging. Circulation journal : official journal of the Japanese Circulation Society. 2007;71(3):363-6. PubMed PMID: 17322636.

29. Sahara M, Kirigaya H, Oikawa Y, Yajima J, Ogasawara K, Satoh H, et al. Arterial remodeling patterns before intervention predict diffuse in-stent restenosis: an intravascular ultrasound study. J Am Coll Cardiol. 2003;42(10):1731-8. PubMed PMID: 14642680.

30. Min JK, Edwardes M, Lin FY, Labounty T, Weinsaft JW, Choi JH, et al. Relationship of coronary artery plaque composition to coronary artery stenosis severity: results from the prospective multicenter ACCURACY trial. Atherosclerosis. 2011;219(2):573-8. doi: 10.1016/j.atherosclerosis.2011.05.032. PubMed PMID: 21696739.

31. Lin F, Shaw LJ, Berman DS, Callister TQ, Weinsaft JW, Wong FJ, et al. Multidetector computed tomography coronary artery plaque predictors of stress-induced myocardial ischemia by SPECT. Atherosclerosis. 2008;197(2):700-9. Epub 2007/08/28. doi: 10.1016/j.atherosclerosis.2007.07.002. PubMed PMID: 17720167.

32. Min JK, Dunning A, Lin FY, Achenbach S, Al-Mallah M, Budoff MJ, et al. Age- and sex-related differences in all-cause mortality risk based on coronary computed tomography angiography findings results from the International Multicenter CONFIRM (Coronary CT Angiography Evaluation for Clinical Outcomes: An International Multicenter Registry) of 23,854 patients without known coronary artery disease. J Am Coll Cardiol. 2011;58(8):849-60. doi: 10.1016/j.jacc.2011.02.074. PubMed PMID: 21835321.

33. Achenbach S, Moselewski F, Ropers D, Ferencik M, Hoffmann U, MacNeill B, et al. Detection of calcified and noncalcified coronary atherosclerotic plaque by contrast-enhanced, submillimeter multidetector spiral computed tomography: a segment-based comparison with intravascular ultrasound. Circulation. 2004;109(1):14-7. doi: 10.1161/01.CIR.0000111517.69230.0F. PubMed PMID: 14691045.

34. Rodriguez-Granillo GA, Agostoni P, Garcia-Garcia HM, Biondi-Zoccai GG, McFadden E, Amoroso G, et al. Meta-analysis of the studies assessing temporal changes in coronary plaque volume using intravascular ultrasound. The American journal of cardiology. 2007;99(1):5-10. doi: 10.1016/j.amjcard.2006.07.054. PubMed PMID: 17196453.

35. Kaneda H, Ako J, Terashima M. Intravascular ultrasound imaging for assessing regression and progression in coronary artery disease. The American journal of cardiology. 2010;106(12):1735-46. doi: 10.1016/j.amjcard.2010.08.012. PubMed PMID: 21126618.

36. Stone GW, Maehara A, Lansky AJ, de Bruyne B, Cristea E, Mintz GS, et al. A prospective natural-history study of coronary atherosclerosis. The New England journal of medicine. 2011;364(3):226-35. doi: 10.1056/NEJMoa1002358. PubMed PMID: 21247313.

37. Syed MA, Oshinski JN, Kitchen C, Ali A, Charnigo RJ, Quyyumi AA. Variability of carotid artery measurements on 3-Tesla MRI and its impact on sample size calculation for clinical research. Int J Cardiovasc Imaging. 2009;25(6):581-9. Epub 2009/05/22. doi: 10.1007/s10554-009-9468-8. PubMed PMID: 19459065.

38. Fan Z, Yu W, Xie Y, Dong L, Yang L, Wang Z, et al. Multi-contrast atherosclerosis characterization (MATCH) of carotid plaque with a single 5-min scan: technical development and clinical feasibility. J Cardiovasc Magn Reson. 2014;16:53. Epub 2014/09/04. doi: 10.1186/s12968-014-0053-5. PubMed PMID: 25184808; PubMed Central PMCID: PMCPMC4222690.

39. Ramadan R, Alkhoder A, Dhawan S, Binongo J, Oshinski J, Quyyumi A. Changes in oxidative stress and vascular function as determinants of progression of atherosclerosis. Journal of the American College of Cardiology. 2014;63(12 Supplement):A2079.

40. Unno N. Utilization of oxidative stress biomarkers is important to assess treatment effects on exercise capacity in patients with intermittent claudication. Circulation Journal. 2014;78(6):1327-8.

41. Mani V, Aguiar SH, Itskovich VV, Weinshelbaum KB, Postley JE, Wasenda EJ, et al. Carotid black blood MRI burden of atherosclerotic disease assessment correlates with ultrasound intima-media thickness. J Cardiovasc Magn Reson. 2006;8(3):529-34. Epub 2006/06/08. doi: 10.1080/10976640600675245. PubMed PMID: 16755842.

42. Sun B, Giddens DP, Long R, Jr., Taylor WR, Weiss D, Joseph G, et al. Characterization of coronary atherosclerotic plaque using multicontrast MRI acquired under simulated in vivo conditions. J Magn Reson Imaging. 2006;24(4):833-41. Epub 2006/08/25. doi: 10.1002/jmri.20687. PubMed PMID: 16929530.

43. Heiss G, Sharrett AR, Barnes R, Chambless LE, Szklo M, Alzola C. Carotid atherosclerosis measured by B-mode ultrasound in populations: associations with cardiovascular risk factors in the ARIC study. Am J Epidemiol. 1991;134(3):250-6. Epub 1991/08/01. doi: 10.1093/oxfordjournals.aje.a116078. PubMed PMID: 1877584.

44. Inaba Y, Chen JA, Bergmann SR. Carotid plaque, compared with carotid intima-media thickness, more accurately predicts coronary artery disease events: a meta-analysis. Atherosclerosis. 2012;220(1):128-33. Epub 2011/07/19. doi: 10.1016/j.atherosclerosis.2011.06.044. PubMed PMID: 21764060.

45. Lorenz MW, Markus HS, Bots ML, Rosvall M, Sitzer M. Prediction of clinical cardiovascular events with carotid intima-media thickness: a systematic review and meta-analysis. Circulation. 2007;115(4):459-67. Epub 2007/01/24. doi: 10.1161/CIRCULATIONAHA.106.628875. PubMed PMID: 17242284.

46. Al Mheid I, Patel R, Murrow J, Morris A, Rahman A, Fike L, et al. Vitamin D status is associated with arterial stiffness and vascular dysfunction in healthy humans. J Am Coll Cardiol. 2011;58(2):186-92. Epub 2011/07/02. doi: 10.1016/j.jacc.2011.02.051. PubMed PMID: 21718915; PubMed Central PMCID: PMCPMC3896949.

47. Topel ML, Hayek SS, Ko YA, Sandesara PB, Samman Tahhan A, Hesaroieh I, et al. Sex Differences in Circulating Progenitor Cells. J Am Heart Assoc. 2017;6(10). Epub 2017/10/05. doi: 10.1161/JAHA.117.006245. PubMed PMID: 28974500; PubMed Central PMCID: PMCPMC5721840.

48. Samman Tahhan A, Hammadah M, Raad M, Almuwaqqat Z, Alkhoder A, Sandesara PB, et al. Progenitor Cells and Clinical Outcomes in Patients With Acute Coronary Syndromes. Circ Res. 2018;122(11):1565-75. Epub 2018/03/09. doi: 10.1161/CIRCRESAHA.118.312821. PubMed PMID: 29514830; PubMed Central PMCID: PMCPMC5970041.

49. Samman Tahhan A, Hammadah M, Sandesara PB, Hayek SS, Kalogeropoulos AP, Alkhoder A, et al. Progenitor Cells and Clinical Outcomes in Patients With Heart Failure. Circ Heart Fail. 2017;10(8). Epub 2017/08/10. doi: 10.1161/CIRCHEARTFAILURE.117.004106. PubMed PMID: 28790053; PubMed Central PMCID: PMCPMC5809135.

50. Mahar EA, Mou L, Hayek SS, Quyyumi AA, Waller EK. Flow cytometric data analysis of circulating progenitor cell stability. Data Brief. 2017;10:346-8. Epub 2016/12/23. doi: 10.1016/j.dib.2016.11.050. PubMed PMID: 28004026; PubMed Central PMCID: PMCPMC5157704.
