# Supplementary material for: Bone, Brain, Heart study protocol: A resilient nested, tripartite prospective cohort study of the role of estrogen depletion on HIV pathology
Source: PLoS One. 2022 Aug 3;17(8):e0272608. doi: 10.1371/journal.pone.0272608 (PMC9348736; doi:10.1371/journal.pone.0272608)
Supplement: S2 Appendix — (DOCX) [file pone.0272608.s002.docx]

Supplement for Bone, Brain, Heart Study protocol: A resilient nested, tripartite prospective cohort study of the role of estrogen depletion on HIV pathology

# Appendix B: Additional Brain, Bone, Heart (BBH) information

## Inclusion/exclusion criteria

Inclusion criteria for BBH are: women age ≥ 30 years who are either at risk for HIV or living with HIV, had an entry HIV-1 RNA of < 50 copies/mL, are currently on ART with ≥ 2 years of ART, and have creatinine clearance (CrCl) ≥50 mL/min estimated by the Cockcroft-Gault equation[1]. Exclusion criteria are: pregnant or breastfeeding; not fully ambulatory; non-HIV-related active immunological or metabolic bone disorders, including: bone marrow or organ transplantation, osteomalacia, osteosarcoma, Paget’s disease, systemic lupus erythematosus, inflammatory bowel diseases, rheumatoid arthritis; thyroid/parathyroid disorders; any cancers (other than skin cancer) and patients exposed to chemotherapies within the past 2 years; chronic kidney disease (CrCL <50 mL/min); chronic liver disease (Liver function tests (LFTs) >3 X ULN); current drug or alcohol abuse; prior acute coronary syndrome (ACS), cardiac arrest, known coronary artery disease, heart failure, or stroke. Since the Bone and Heart/vascular Projects involve procedures that have additional inclusion/exclusion criteria, consented participants receive additional screening for eligibility. For the cardiovascular magnetic resonance imaging (MRI), additional exclusion criteria were the presence of metal in the body and allergy to contrast. As needed, each Project rescreens for selected relevant time-varying eligibility criteria prior to commencing a procedure.

## Databases

All study-related and participant-related data are collected and stored on a secure, HIPAA-compliant network server. The Shared Inter-Project Participant (SIPP) Database was designed using Microsoft Access to facilitate the construction of customized, complex queries and forms that allow the real-time tracking of information described above. It is also able to automatically extract relevant participant information from a linked WIHS/MWCCS SIPP database, thus eliminating double-entry and saving staff time. By updating and accessing the SIPP Database on a real-time basis, study personnel for each Project are able to stay up to date on shared-participant study progress.

To address the complexity imposed by the Study’s tripartite nature, BBH staff created a Research Electronic Data Capture (REDCap) Database to capture BBH project-specific data, the BBH Outcomes Database. REDCap is a secure, web-based software platform designed to support data capture for research studies[2, 3]. The REDCap BBH Outcomes Database provides real-time shared access for all Project staff conducting participant interviews and concurrent data entry. As an approved sub-study of the WIHS/MWCCS, the necessary information collected from WIHS/MWCCS core visits will be extracted for the linked ID-visits for BBH participants using the closest previous core visit corresponding to each BBH visit, as appropriate. An analytic dataset for BBH will be created based on a merge of the BBH Outcomes Database with the WIHS/MWCCS extracted data.

## Participant safety

Prior to study initiation, protocols regarding clinically urgent findings were put in place that prompt immediate action and referral when needed to ensure the safety of all participants. Other protocols were established that initiate the notification of a participant’s primary care provider when needed for timely, but non-urgent, clinical follow-up needs that have been identified during a study visit.

## Quality control

Monthly, Project-specific quality assessment reports about the clinical, interview, and imaging data entered into the BBH Outcomes Database are provided for each Project and the overall BBH sub-study. Additional reports assess study completion across Projects based on queries in the SIPP Database.

# Statistical analysis plan

The study sample, both overall and by HIV status, will be described by counts and frequencies for categorical variables, mean and standard deviation for normally distributed continuous variables and median, quartile 1 and quartile 3 for non-normal continuous variables. Chi-square tests, Fisher exact tests, t-tests, and Wilcoxon rank sum tests, as appropriate, will test for association with HIV status. Distributional assumptions will be checked. If assumptions are violated, alternative methods such as data transformations (e.g., natural log) or the use of nonparametric techniques will be used. For all models: appropriate regression diagnostics will be performed including: consideration of model overfitting, assessment of collinearity, Hosmer-Lemeshow goodness of fit statistics, discriminatory ability, and examination of residual plots; if model assumptions are not met, transformations or alternative approaches will be used. Outlines of the analytic plans for each aim are described below. All analyses will be conducted in SAS v9.4.

## Project 1: Brain

Aim 1

Due to likely skewness cytokine data (IL-6, TNFα, and IL-1β) will be log transformed prior to analysis. Known confounders associated with inflammation include age, drug use, smoking status, PTSD, and depression. The main estrogen marker of interest is gene expression of ERα. Variables significant at the p<0.1 level in bivariate analysis and known confounders and other covariates will be explored for inclusion in the final multivariable models. Because primary interest is in the relationship between inflammatory cytokines and estrogen markers, covariate inclusion will be based on model fit and adequate control of confounding. Separate linear regression models will assess the relationship between each cytokine (IL-6, TNFα, and IL-1β) and each estrogen marker (estradiol; AMH; ERα, ERβ expression), controlling for relevant covariates. Because each estrogen marker measures a unique aspect of estrogen deficiency, model results will focus on the pattern and clinical importance of results and not statistical significance except for gene expression of ERα which will be assessed at a significance level of 0.05.

Aim 2

Trauma scale scores will be calculated according to previously published instructions. The main cytokine of interest is TNFα, the main estrogen marker of interest is estradiol, and the main predictors of interest are SC and TEI. Models of these main outcomes and main trauma variable will be assessed at a significance level of 0.05. Separate linear regression models will assess the relationship between each cytokine (IL-6, TNFα, IL-1β) and each estrogen marker (estradiol; ERα, ERβ expression) by HIV status and trauma measure (TEI, CTQ, SC), controlling for relevant covariates (CAPS, MINI). All models will include HIV, trauma measure, and an HIV*trauma measure interaction terms. Because each estrogen marker measures a unique aspect of estrogen deficiency and each cytokine measures a unique aspect of inflammation, model results will focus on pattern and clinical importance of results and not statistical significance, except for models of TNFα and estradiol with the trauma measures of SC and TEI.

Aim 3

Bivariate analysis will compare groups by inflammation group (top or bottom tertile). In order to incorporate repeated measures, all models will use Generalized Estimating Equations (GEE), assume a normal distribution, and assume a compound symmetry covariance matrix. To evaluate the ER response to estradiol, separate GEE models will be used to assess gene expression of 84 ER-regulated genes with inflammation group and trauma measure. Models will contain inflammation group, trauma measure (TEI, CTQ, CS) and type of gene (ER-regulated, housekeeping) and all interaction terms. To evaluate ER response to inflammatory stimulus, separate GEE models will be used to assess ER expression (ERα, ERβ) with treatment condition (0 ng/ml LPS, 30 ng/ml LPS). Models will contain inflammation group, treatment condition, trauma measure, and all interaction terms. To evaluate ER subtype modulation of inflammatory stimulus, a GEE model will be used to assess ER expression (ERα or ERβ) by treatment (control, estradiol, agonist (ERα or ERβ), dexamethasone), inflammation group, and trauma measure. Models will contain inflammation group, treatment, trauma measure, all interaction terms. Because each of these analyses measures a unique aspect of ER function, model results will focus on pattern and clinical importance of results and not statistical significance, except for models of ERα which will be evaluated at a significance level of 0.05.

## Project 2: Bone

Aim 1

Individual linear regression models will assess the association of each outcome (PBMC production of OPG, RANKL, TNFα; BMD) with HIV status and estrogen level). Models will include HIV status, estrogen level, and an HIV*estrogen interaction term. HIV-only models will assess relationship between estrogen level after adjusting for HIV-specific covariates such as ART adherence and type of ART. However, primary interest is in the interaction of HIV with estrogen levels on the outcome of hip BMD, (a common and devastating site for fracture) which will be evaluated with a significance level of 0.05. The magnitude, clinical meaningfulness, and pattern of results of the remaining models will be evaluated instead of statistical significance. Models will additionally contain covariates known to be related to the outcomes including age and race.

Aim 2

Simple comparisons between groups involve 2-sample t-test or Kruskal-Wallis if non-parametric. Multiple comparisons by one-way ANOVA or repeated measures ANOVA if prospective. Non-parametric tests involve Kruskal-Wallis for one-way ANOVA or Dunn's test for repeated measures ANOVA. Significance level of α=0.05 for all tests.

## Project 3: Heart/vessels

Aim 1

Covariates of interest include age, BMI, estrogen level, diabetes, hypertension, dyslipidemia, and other traditional cardiovascular risk factors. The main PC subtype biomarker of interest is the CD34+ cell count and the main coronary outcomes are presence of coronary plaque and its severity; the main carotid outcomes of interest are presence of carotid plaque and mean wall thickness (WT). Additional bivariate tests will assess for significant associations between coronary and carotid plaque presence and covariates. Variables significant at the p<0.1 level in bivariate analysis and known confounders and other covariates will be explored for inclusion in the final multivariable models. Since Primary Aim 1 is on the association of HIV status and PC level with coronary and carotid disease, final covariate inclusion will be based on model fit and adequate control of confounding. Coronary plaque is defined as any tissue >1mm^2^ within or adjacent to the lumen that can be identified in >2 planes[4]. The angiographic CAD extent and severity will be measured using the SCCT 5-point scale[5]. Carotid plaque is defined as a maximum WT >2 mm[6].

For the main analysis of Primary Aim 1a, separate multivariable regression models will evaluate the association of HIV status with CD34+ PC count on presence (using a logistic model) and severity of coronary plaque (using an ordinal logistic model), controlling for important covariates. The model will include HIV status, PC count, and an HIV status*PC count interaction term. The main analysis for Aim 1b will use two similar multivariable regression models to evaluate the association of HIV status and CD34+ PC count on carotid plaque presence (logistic regression model) and mean WT (linear regression model), controlling for important covariates. The significance level for these models is set to 0.05. Additionally, for Aim 1a similar separate multivariable (linear or ordinal as appropriate) regression models to evaluate the association of HIV status and other PC sub-populations (CD34+/CD133+, CD34+/VEGF2R+, and CD34+/CXCR4) and each of the coronary and carotid outcomes above. Lastly, all models will be repeated with WLH only, in order to adjust for the effect of HAART use and adherence, CD_4_+ cell count, HIV-1 RNA on the outcomes.

We will use the same methodology for Secondary Aim 1 to separately assess the association of HIV status and each of endothelial function (FMD) and arterial stiffness (PWV, AIx) measures on each outcome. Since each outcome assessed for Aim 1 measures a unique aspect of CVD, model results will focus on the pattern and clinical importance of results and not statistical significance except for presence of coronary plaques and presence of carotid plaques.

### Aim 2

For Primary Aim 2, the primary models of interest are linear mixed effect models that will assess association of change in each primary endpoint (VWA, WT) from baseline to 2 years with HIV status and time point. The model will include HIV status, time (baseline, 2 year follow-up), and HIV*time interaction term. Models will include traditional CVD risk factors and other potential confounders (as described in Aim 1), and use an unstructured covariance matrix and a random intercept. The significance level for these models will be set at 0.05. Model fit will be evaluated through residual plots and assessment of random and fixed effect terms.

For Secondary Aim 2, similar separate linear mixed effect models will assess change in each primary endpoint (VWA, WT) from baseline to 2 years by HIV status, time, and each independent variable of interest (PC biomarkers (CD34+, CD34+/CD133+, CD34+/VEGF2R+, CD34+/CXCR4 counts), markers of inflammation (hsCRP, TNFα, IL-6, IL-1β), vascular dysfunction (FMD, PWV, AIx), estrogen levels). The model will include HIV status, time, independent variable of interest, and all interaction terms. Similarly, logistic mixed effect models will be used to assess the association of change in presence of carotid plaque over time by HIV status and also by HIV status and each of the independent variables of interest. Since these models investigate the moderation of the endpoints by HIV and different factors (PCs, markers of inflammation, vascular dysfunction, estrogen levels), interpretation of model results will focus on the pattern and clinical importance of results and not statistical significance.

Aim 3

For Primary Aim 3, bivariable analysis (t-tests/ANOVA, correlation) will assess the relationship between total atherosclerotic plaque burden and traditional cardiovascular risk factors and covariates (described in Aim 1) and also cocaine or intravenous drug use, income, educational level, unstable housing situation, regularity of healthcare use, and social support variables. Socioeconomic and psychosocial factors (i.e., depression or stress) may also be considered as they have been previously shown to risk stratify women[7]. Variables significant at the 0.10 level, variables that were significantly different by HIV status (assessed in Aim 1), and known confounders (such as traditional CAD risk factors: BMI, age, diabetes, dyslipidemia, hypertension) will be considered for inclusion in the model. A multivariable linear regression model will evaluate the association of HIV status with total atherosclerotic plaque burden, controlling for important covariates. Since primary interest is in the association of HIV status with total atherosclerotic plaque burden, final covariate inclusion will be based on model fit and adequate control of confounding. For Secondary Aim 3, a similar multivariable regression model will evaluate the association of HIV status and estrogen level (continuous) on total atherosclerotic plaque burden, controlling for the important covariates from Primary Aim 3. Significance level for these models will be set at 0.05. Additionally, for Exploratory Aim 3 multivariable (linear or ordinal as appropriate) regression models will evaluate the association of HIV status and individual high risk plaque characteristics (lowest attenuation plaque (linear), remodeling (ordinal), plaque composition (ordinal), plaque burden (linear), minimal luminal area (linear)) and HRP extent (ordinal). Since this aim is exploratory, interpretation of model results will focus on the pattern and clinical importance of results and not statistical significance. Models with WLH only, will consider HAART use and adherence, CD_4_+ cell count, HIV-1 RNA. Similar approaches to model development will be applied as noted previously.

# References

1. Cockcroft DW, Gault MH. Prediction of creatinine clearance from serum creatinine. Nephron. 1976;16(1):31-41. Epub 1976/01/01. doi: 10.1159/000180580. PubMed PMID: 1244564.

2. Harris PA, Taylor R, Minor BL, Elliott V, Fernandez M, O'Neal L, et al. The REDCap consortium: Building an international community of software platform partners. J Biomed Inform. 2019;95:103208. Epub 2019/05/13. doi: 10.1016/j.jbi.2019.103208. PubMed PMID: 31078660; PubMed Central PMCID: PMCPMC7254481.

3. Harris PA, Taylor R, Thielke R, Payne J, Gonzalez N, Conde JG. Research electronic data capture (REDCap)--a metadata-driven methodology and workflow process for providing translational research informatics support. J Biomed Inform. 2009;42(2):377-81. Epub 2008/10/22. doi: 10.1016/j.jbi.2008.08.010. PubMed PMID: 18929686; PubMed Central PMCID: PMCPMC2700030.

4. Budoff MJ, Dowe D, Jollis JG, Gitter M, Sutherland J, Halamert E, et al. Diagnostic performance of 64-multidetector row coronary computed tomographic angiography for evaluation of coronary artery stenosis in individuals without known coronary artery disease: results from the prospective multicenter ACCURACY (Assessment by Coronary Computed Tomographic Angiography of Individuals Undergoing Invasive Coronary Angiography) trial. J Am Coll Cardiol. 2008;52(21):1724-32. Epub 2008/11/15. doi: 10.1016/j.jacc.2008.07.031. PubMed PMID: 19007693.

5. Raff GL, Abidov A, Achenbach S, Berman DS, Boxt LM, Budoff MJ, et al. SCCT guidelines for the interpretation and reporting of coronary computed tomographic angiography. J Cardiovasc Comput Tomogr. 2009;3(2):122-36. Epub 2009/03/11. doi: 10.1016/j.jcct.2009.01.001. PubMed PMID: 19272853.

6. Mani V, Aguiar SH, Itskovich VV, Weinshelbaum KB, Postley JE, Wasenda EJ, et al. Carotid black blood MRI burden of atherosclerotic disease assessment correlates with ultrasound intima-media thickness. J Cardiovasc Magn Reson. 2006;8(3):529-34. Epub 2006/06/08. doi: 10.1080/10976640600675245. PubMed PMID: 16755842.

7. Shaw LJ, Merz CN, Bittner V, Kip K, Johnson BD, Reis SE, et al. Importance of socioeconomic status as a predictor of cardiovascular outcome and costs of care in women with suspected myocardial ischemia. Results from the National Institutes of Health, National Heart, Lung and Blood Institute-sponsored Women's Ischemia Syndrome Evaluation (WISE). J Womens Health (Larchmt). 2008;17(7):1081-92. doi: 10.1089/jwh.2007.0596. PubMed PMID: 18774893; PubMed Central PMCID: PMCPMC2818766.
