# Supplementary material for: Bone, Brain, Heart study protocol: A resilient nested, tripartite prospective cohort study of the role of estrogen depletion on HIV pathology
Source: PLoS One. 2022 Aug 3;17(8):e0272608. doi: 10.1371/journal.pone.0272608 (PMC9348736; doi:10.1371/journal.pone.0272608)
Supplement: S3 Appendix — (DOCX) [file pone.0272608.s003.docx]

Supplement for Bone, Brain, Heart Study protocol: A resilient nested, tripartite prospective cohort study of the role of estrogen depletion on HIV pathology

Appendix C. Additional protocol information for animal subjects

Project 2: Bone, Aim 2 protocol was approved by the Emory University Institutional Animal Care and Use Committee (IACUC, Protocol Approval # PROTO201800168).

## Steps taken to ameliorate animal suffering

In vivo prospective imaging procedures such as DEXA to quantify BMD, or micro-computed tomography to quantify bone volume, may be performed at baseline and at regular intervals of 2 to 4 weeks thereafter, for the duration of the study. These procedures are inherently non-invasive and are not associated with any pain. However, mice need to be restrained during the scans, which would be stressful to the animals and is hence performed under isoflurane anesthesia (2.5% Isoflurane in 100% oxygen) delivered using a vaporizer and gas delivered through a rodent mask placed over the nose/mouth.

T cell adoptive transfer may be performed by tail vein injection or retro-orbital injection. A mouse restrainer tube is used for immobilization for tail vein injection and these procedures cause only momentary discomfort. Retro-orbital injection is performed under isoflurane anesthesia (2-3%) in order to alleviate distress of handling.

Ovariectomy and sham surgery is performed in anesthetized mice (2.5% Isoflurane in 100% oxygen) delivered using a vaporizer and gas delivered through a rodent mask placed over the nose/mouth. There is a potential for a limited amount of post-surgical pain associated with the incision and mice receive analgesic (Meloxicam (5 mg/kg SC or IP) immediately prior to surgery and then every 24 hours for 2 days to alleviate post-surgical pain.

## Humane endpoints

Emory University IACUC default endpoints will be used to deal with any animals that unexpectedly become sick or moribund and for rodents include:

1. Loss of 25% of body weight from baseline weight when assigned to the protocol and using a growth nomogram to adjust the basal weight for growing animals.
2. Major organ failure or medical conditions unresponsive to treatment such as respiratory distress, icterus, uremia, intractable diarrhea, or self-mutilation.
3. Surgical complications unresponsive to immediate intervention; i.e. bleeding, vascular graft/circulation failure, infection, and wound dehiscence.
4. Clinical or behavioral signs unresponsive to appropriate intervention persisting for 24 hours including: inactivity, labored breathing, sunken eyes, hunched posture, piloerection/matted fur, one or more unresolving skin ulcers, abnormal vocalization when handled, tumors that affect normal function or that become ulcerated, and anorexia.

Any mouse displaying default endpoints will be assumed to be sick and in pain and sacrificed within 24 hours, if no remedy is available after consultation with the Emory veterinarians.
