# Supplementary material for: Bone, Brain, Heart study protocol: A resilient nested, tripartite prospective cohort study of the role of estrogen depletion on HIV pathology
Source: PLoS One. 2022 Aug 3;17(8):e0272608. doi: 10.1371/journal.pone.0272608 (PMC9348736; doi:10.1371/journal.pone.0272608)
Supplement: S1 Table — (DOCX) [file pone.0272608.s004.docx]

Supplement for Bone, Brain, Heart Study protocol: A resilient nested, tripartite prospective cohort study of the role of estrogen depletion on HIV pathology

**S1 Table. Power calculations for each specific aim of the Brain, Bone, Heart (BBH) Study Projects**

| **Project** | **Aim** |
| --- | --- |
| Project 1: Brain | Aim 1. Primary outcome: TNF-α  A study of 130 osteoarthritic women found a correlation of -0.4 between estrogen and TNF-α[1]. Because our sample of MWCCS women is more diverse, we assume a slightly less strong correlation of -0.3. A sample size of 261 women achieves 80% power to detect a difference of 0.15 between the null hypothesis Pearson correlation of -0.3 between TNF-α and estrogen and the alternative hypothesis correlation of -0.45 using a two-sided hypothesis test with a significance level of 0.05. |
|  | Aim 2. Primary outcome: Estradiol  A study of 81 women examined estrogen, trauma exposure, Post-traumatic stress disorder (PTSD), and hyperarousal[2]. Their findings show increased hyperarousal (45uV±8; n=41) in women with PTSD is associated with mean estradiol level of 8 pg/ml, whereas low hyperarousal (10uV±10; n=15) in women with PTSD is associated with a mean of 92.5 pg/ml. We propose that HIV infection will exacerbate the effects of trauma and hyperarousal on estradiol levels and inflammation. Assuming a common SD of 40 for estradiol, estradiol to be 50 pg/ml for HIV negative women, group sample sizes of n=87 HIV negative women and n=174 WLH achieves 81% power to reject the null hypothesis of equal mean estradiol level when the population mean difference is 65-50=15 with a significance level of 0.05 using a two-sided two-sample equal-variance t-test. |
|  | Aim 3 (exploratory). Primary outcome: TNF-α  Previous data suggest the percent dexamethasone suppression to lipopolysaccharide (LPS) induced TNF-α to be approximately 50 in HIV negative participants and 55 in HIV positive participants with a common SD of 8. We expect that incorporating trauma will increase the differences between groups, thus group sample sizes of 50 WLH and 50 HIV negative women achieves 87 power to reject the null hypothesis of equal means when the population mean difference 50 - 55 = -5 with a significance level of 0.05 using a two-sided two-sample equal-variance t-test. |
| Project 2: Bone | Aim 1. Primary outcome: total hip bone mineral density (BMD)  BMD varies by race group in the general population, so BMD calculations are weighted by the race distribution observed among women in the Atlanta MWCCS cohort (~90% African-American). For these calculations age categories of 35 years (peak BMD) and 55 years are used as a proxy for menopause status (Mean age of menopause = 51 in HIV^-^ women. Based on NHANES data we used a mean BMD for HIV^-^, pre-menopause women of 1064 mg/cm^2^ and mean BMD of 894 mg/cm^2^for HIV negative, post-menopausal women 10 years after the menopause (65 years), an average of 16% loss in BMD the end of the of high bone loss phase. For pre-menopausal black WLH we used empirical data corrected for race distribution with mean BMD of 999 mg/cm^2^. Since we are predicting at least an additive effect of HIV/ART and estrogen decline, we predict a mean decline to 839 mg/cm^2^ compared to HIV^-^, women by age 65. We further used a common SD of 129 and a significance level of 0.05. Group sample sizes of 30 HIV negative pre-menopause, 30 HIV negative post-menopause, 60 pre-menopause WLH, and 60 post-menopause WLH (total n=180) achieves 80% power (95% CI 78%, 83%) to detect an HIV status*menopause status interaction of 55 mg/cm^2^ using n=1000 regression model simulations. |
|  | Aim 2*. Primary outcome: Bone Volume Fraction  Group size = 12 mice/group. Using pilot 𝜇CT data we calculated the power of a two-tailed 1-Sample Z-test to detect a significant difference in the key 𝜇CT index Bone Volume Fraction between T cell receptor (TCR) 𝛽 knock-out (KO) mice and TCR𝛽 KO + T cells with maximum SD of 2.25 and power > 0.95 to detect a difference in Bone Volume Fraction of 1.92. |
| Project 3: Heart/vascular | Aim 1 Part A. Primary outcome: coronary plaque prevalence  Aim 1 Part B. Primary outcome: carotid mean wall thickness averaged over circumferential locations (WT)  Preliminary data suggest progenitor cell counts are lower and WT higher in WLH compared to HIV- women. Fadini et al, reported significant correlation (r=-0.277) between CD34+/VEGF+ PC count and CIMT in the general population, and Verma et al found a correlation (r=-0.767) between the CD34+/CD133+ PC count and CIMT in ankylosing spondylitis, thus we expect WLH to have a correlation between that range and therefore have much higher CIMT than HIV- women[3, 4] A sample size of 60 HIV- and 120 WLH achieves 99% power to reject the null hypothesis of equal mean carotid WT when the population mean difference is 0.1 with a common SD of 0.14 and a significance level of 0.05 using a two-sided two sample equal variance t-test.  The prevalence coronary plaque in WLH is approximately 35% and 15% in HIV- women (CONFIRM registry). We expect larger differences in coronary plaque prevalence due to the difference in PC levels between the groups. Thus, a sample size of n=60 HIV- and n=120 WLH achieves 98% power to detect a difference between the group proportions of -0.3 where proportion is assumed to be 0.45 under the null hypothesis and 0.15 under the alternative hypothesis using a two-sided Fisher's Exact Test and a significance level of 0.05. |
|  | Aim 2. Primary outcome: change in Vessel Wall Area (VWA)  Preliminary data show that the SD is 3.1 for change from baseline to 2 years in VWA, 0.08 for change in WT[5]. Assuming 20% dropout and a significance level of 0.05, an original sample size of 120 WLH and 60 in the HIV- group will achieve 90% power to detect a difference of 1.78 in change in VWA using an independent sample t-test. Similarly, a 0.046 in change in WT achieves 90% power. The minimum detectable group differences in this aim are smaller than the observed differences by HIV status in our preliminary data. |
|  | Aim 3. Primary outcome: Total atherosclerotic plaque  Previous research suggests that most adults (age >40 years) have some atherosclerotic plaque present. The total atherosclerotic plaque in a group with a high risk factor burden is approximately 189 mm3 (SD 189) compared to a group with no risk factors which has a mean of 90 mm3 (SD 90) (CONFIRM registry). Assuming HIV infection is similar to having a lot of risk factors, setting the significance level to 0.05, n=60 HIV negative and n=120 WLH achieves 98% power to detect a 189-99=90 difference in total atherosclerotic plaque using an independent samples t-test with unequal variances. |

* Conducted in mouse model

Legend: WLH: women living with HIV; SD: standard deviation; CIMT: Carotid intima-media thickness; TNF-α: tumor necrosis factor-α,

All power calculations were conducted in PASS.

# References

1. Sharma P, Singh N, Singh V, Singh S, Singh HV, Gupta S. Tumor necrosis factor alpha (TNF-alpha) and estrogen hormone in osteoarthritic female patients. Indian J Clin Biochem. 2006;21(1):205-7. Epub 2006/03/01. doi: 10.1007/BF02913099. PubMed PMID: 23105602; PubMed Central PMCID: PMCPMC3453781.

2. Glover EM, Jovanovic T, Mercer KB, Kerley K, Bradley B, Ressler KJ, et al. Estrogen levels are associated with extinction deficits in women with posttraumatic stress disorder. Biol Psychiatry. 2012;72(1):19-24. Epub 2012/04/17. doi: 10.1016/j.biopsych.2012.02.031. PubMed PMID: 22502987; PubMed Central PMCID: PMCPMC3675159.

3. Verma I, Syngle A, Krishan P, Garg N. Endothelial Progenitor Cells as a Marker of Endothelial Dysfunction and Atherosclerosis in Ankylosing Spondylitis: A Cross-Sectional Study. Int J Angiol. 2017;26(1):36-42. Epub 2017/03/04. doi: 10.1055/s-0036-1593445. PubMed PMID: 28255214; PubMed Central PMCID: PMCPMC5330760.

4. Fadini GP, Coracina A, Baesso I, Agostini C, Tiengo A, Avogaro A, et al. Peripheral blood CD34+KDR+ endothelial progenitor cells are determinants of subclinical atherosclerosis in a middle-aged general population. Stroke. 2006;37(9):2277-82. Epub 2006/07/29. doi: 10.1161/01.STR.0000236064.19293.79. PubMed PMID: 16873710.

5. Ramadan R, Dhawan SS, Binongo JN, Alkhoder A, Jones DP, Oshinski JN, et al. Effect of Angiotensin II Type I Receptor Blockade with Valsartan on Carotid Artery Atherosclerosis: A Double Blind Randomized Clinical Trial Comparing Valsartan and Placebo (EFFERVESCENT). Am Heart J. 2016;174:68-79. Epub 2016/03/21. doi: 10.1016/j.ahj.2015.12.021. PubMed PMID: 26995372; PubMed Central PMCID: PMCPMC4800491.
