# Supplementary material for: Bone, Brain, Heart study protocol: A resilient nested, tripartite prospective cohort study of the role of estrogen depletion on HIV pathology
Source: PLoS One. 2022 Aug 3;17(8):e0272608. doi: 10.1371/journal.pone.0272608 (PMC9348736; doi:10.1371/journal.pone.0272608)
Supplement: S2 Table — (DOCX) [file pone.0272608.s005.docx]

Supplement for Bone, Brain, Heart Study protocol: A resilient nested, tripartite prospective cohort study of the role of estrogen depletion on HIV pathology

**S2 Table: Brain, Bone, Heart (BBH) Study STROBE[1] statement checklist**

|  | Item No. | Recommendation | | | | Page  No. | | | Relevant text from manuscript | | |
| --- | --- | --- | --- | --- | --- | --- | --- | --- | --- | --- | --- |
| **Title and abstract** | 1 | (a) Indicate the study’s design with a commonly used term in the title or the abstract | | | | 1 (title), 2 (abstract) | | | “nested, tripartite, prospective observational cohort study” |  |  |
|  |  | (b) Provide in the abstract an informative and balanced summary of what was done and what was found | | | | 2 | | | Abstract Methods and Conclusion sections |  |  |
| Introduction | | | | | | | | |  | | |
| Background/rationale | 2 | | Explain the scientific background and rationale for the investigation being reported | | | 3-4 | | | Sections on: Estrogen deficiency and immune activation; immune effects of HIV infection; previous research on estrogen deficiency, HIV infection and brain, bone, heart outcomes | | |
| Objectives | 3 | | State specific objectives, including any pre-specified hypotheses | | | 4-5; 12-13 | | | Overall research aim; Specific Aims described in Table 1 | | |
| Methods | | | | | | | | |  | | |
| Study design | 4 | | Present key elements of study design early in the paper | | | 5 | | | Study design and participants section | | |
| Setting | 5 | | Describe the setting, locations, and relevant dates, including periods of recruitment, exposure, follow-up, and data collection | | | 5-7 | | | Sections on: Study design and participants; Data collection; Contingencies | | |
| Participants | 6 | | (a) *Cohort study*—Give the eligibility criteria, and the sources and methods of selection of participants. Describe methods of follow-up | | | 5, Supplement | | | Sections on: Study design and participants; Appendix B: Inclusion/exclusion criteria | | |
| Variables | 7 | | | Clearly define all outcomes, exposures, predictors, potential confounders, and effect modifiers. Give diagnostic criteria, if applicable | | Supplement 1-8 | | | Appendix A | | |
| Data sources/ measurement | 8 | | | For each variable of interest, give sources of data and details of methods of assessment (measurement). Describe comparability of assessment methods if there is more than one group | | Supplement 1-8 | | | Appendix A | | |
| Bias | 9 | | | Describe any efforts to address potential sources of bias | | 6 | | | Data collection section |  |  |
| Study size | 10 | | | Explain how the study size was arrived at | | Supplement Table 1 | | | Power calculations for each Project and aim |  |  |
| Quantitative variables | 11 | | | | Explain how quantitative variables were handled in the analyses. If applicable, describe which groupings were chosen and why | N/A | | |  |  |  |
| Statistical methods | 12 | | | | (a) Describe all statistical methods, including those used to control for confounding | N/A | | |  |  |  |
|  |  |  |  |  | (b) Describe any methods used to examine subgroups and interactions | N/A | | |  |  |  |
|  |  |  |  |  | (c) Explain how missing data were addressed | N/A | | |  |  |  |
|  |  |  |  |  | (d) *Cohort study*—If applicable, explain how loss to follow-up was addressed | 7 | | | Logistics section |  |  |
|  |  |  |  |  | (e) Describe any sensitivity analyses | N/A | | |  |  |  |
| Results | | | | | | | | | |  |  |
| Participants | 13 | | | | (a) Report numbers of individuals at each stage of study—eg numbers potentially eligible, examined for eligibility, confirmed eligible, included in the study, completing follow-up, and analysed | N/A | |  | | | |
|  |  |  |  |  | (b) Give reasons for non-participation at each stage | N/A | |  | | | |
|  |  |  |  |  | (c) Consider use of a flow diagram | N/A | |  | | | |
| Descriptive data | 14 | | | | (a) Give characteristics of study participants (eg demographic, clinical, social) and information on exposures and potential confounders | N/A | |  | | | |
|  |  |  |  |  | (b) Indicate number of participants with missing data for each variable of interest | N/A | |  | | | |
|  |  |  |  |  | (c) *Cohort study*—Summarise follow-up time (eg, average and total amount) | N/A | |  | | | |
| Outcome data | 15 | | | | *Cohort study*—Report numbers of outcome events or summary measures over time | N/A | |  | | | |
| Main results | 16 | | | | (a) Give unadjusted estimates and, if applicable, confounder-adjusted estimates and their precision (eg, 95% confidence interval). Make clear which confounders were adjusted for and why they were included | N/A | |  | | | |
|  |  |  |  |  | (b) Report category boundaries when continuous variables were categorized | N/A | |  | | | |
|  |  |  |  |  | (c) If relevant, consider translating estimates of relative risk into absolute risk for a meaningful time period | N/A | |  | | | |
| Other analyses | 17 | | | | Report other analyses done—eg analyses of subgroups and interactions, and sensitivity analyses | N/A |  | | | |  |
| Discussion | | | | | | | | | | |  |
| Key results | 18 | | | | Summarise key results with reference to study objectives | N/A |  | | | |  |
| Limitations | 19 | | | | Discuss limitations of the study, taking into account sources of potential bias or imprecision. Discuss both direction and magnitude of any potential bias | N/A |  | | | |  |
| Interpretation | 20 | | | | Give a cautious overall interpretation of results considering objectives, limitations, multiplicity of analyses, results from similar studies, and other relevant evidence | N/A |  | | | |  |
| Generalisability | 21 | | | | Discuss the generalisability (external validity) of the study results | N/A |  | | | |  |
| Other information |  | | | | | | | | | |  |
| Funding | 22 | | | | Give the source of funding and the role of the funders for the present study and, if applicable, for the original study on which the present article is based | 9 | Acknowledgements | | | |  |

# References

1. von Elm E, Altman DG, Egger M, Pocock SJ, Gotzsche PC, Vandenbroucke JP, et al. The Strengthening the Reporting of Observational Studies in Epidemiology (STROBE) statement: guidelines for reporting observational studies. Epidemiology. 2007;18(6):800-4. Epub 2007/12/01. doi: 10.1097/EDE.0b013e3181577654. PubMed PMID: 18049194.
